# Supplementary figures and images for: Post-translational modifications glycosylation and phosphorylation of the major hepatic plasma protein fetuin-A are associated with CNS inflammation in children
Source: PLoS One. 2022 Oct 7;17(10):e0268592. doi: 10.1371/journal.pone.0268592 (PMC9544022; doi:10.1371/journal.pone.0268592)

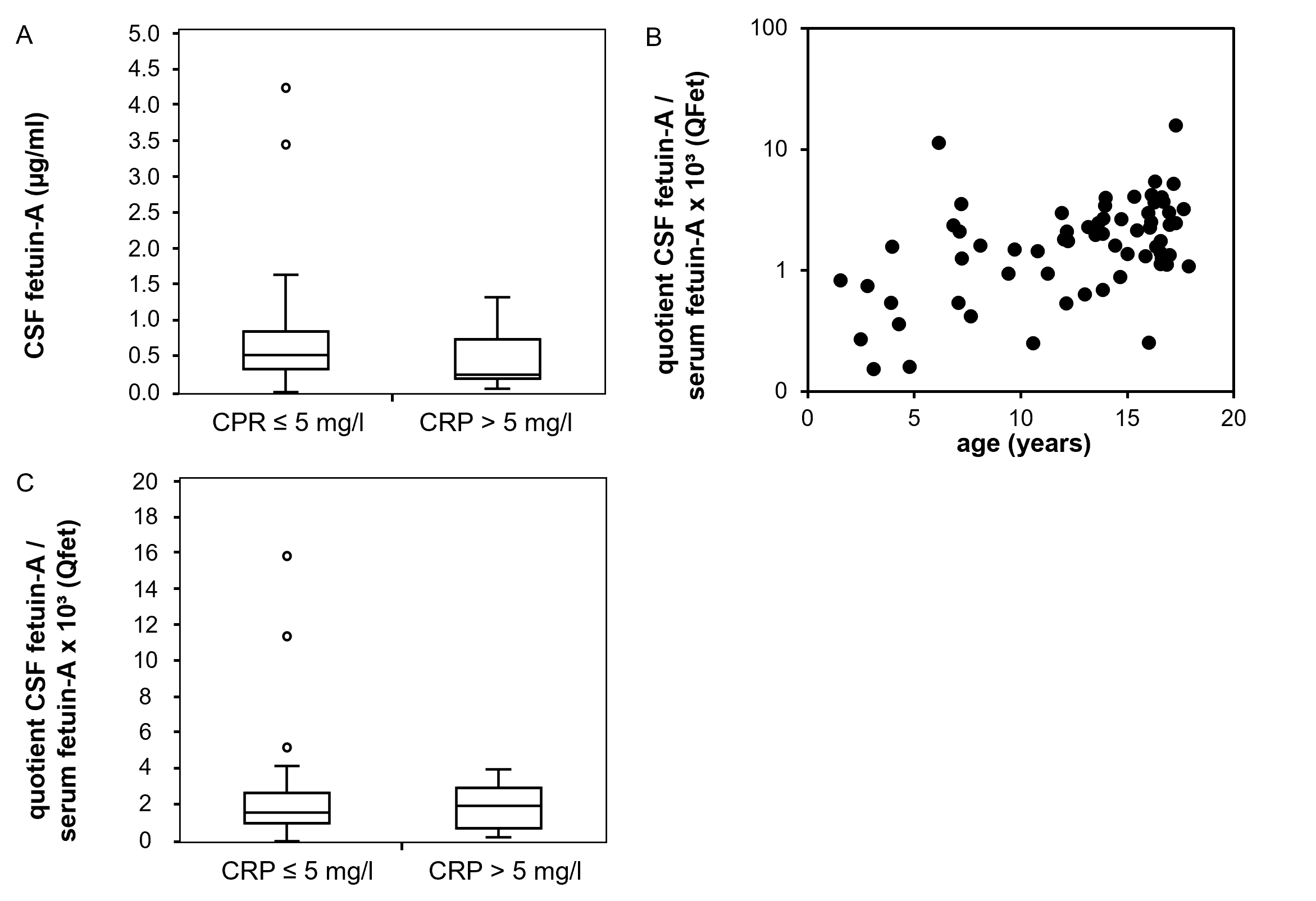

Supplement: S1 Fig — shows supplementary results of the concentrations measurements of fetuin-A in CSF and serum. The correlation of CSF fetuin-A (y-axis, μg/ml) with an elevated C-reactive protein concentration (x-axis) is displayed as a boxplot in (A). The connection between the CSF fetuin-A/serum fetuin-A quotient (y-axis, x103) and age (x-axis, years) is displayed as scatter plot in (B). (C) shows the correlation of the CSF fetuin-A/serum fetuin-A quotient (y-axis, x103) with an elevated C-reactive protein concentration (x-axis) as a boxplot. (TIF) [file pone.0268592.s001.tif]
