# Supplementary material for: Post-translational modifications glycosylation and phosphorylation of the major hepatic plasma protein fetuin-A are associated with CNS inflammation in children
Source: PLoS One. 2022 Oct 7;17(10):e0268592. doi: 10.1371/journal.pone.0268592 (PMC9544022; doi:10.1371/journal.pone.0268592)
Supplement: S1 Table — Predictors for fetuin-A concentration (mg/ml) in serum. (PDF) [file pone.0268592.s002.pdf]

S1 Table: Multiple linear regression. Predictors for fetuin-A concentration (mg / ml) in serum

**Model summary**	**Adjusted R^2**				
	0.215				
**ANOVA**	**F (2,57)**	**p**			
	9.06	P<0.001			
**Model**	**B***	**Beta****	**Signifi-cance**	**CI for B low**	**CI for B high**
Constant	0.337		0.000	0.314	0.359
C-reactive protein level (normal / increased)	-0.069	-0.386	0.001	-0.11	-0.028
Inflammatory CNS disease (no / yes)	-0.041	-0.33	0.006	-0.07	-0.012

* unstandardized coefficients. ** standardized coefficients
