# Supplementary material for: Post-translational modifications glycosylation and phosphorylation of the major hepatic plasma protein fetuin-A are associated with CNS inflammation in children
Source: PLoS One. 2022 Oct 7;17(10):e0268592. doi: 10.1371/journal.pone.0268592 (PMC9544022; doi:10.1371/journal.pone.0268592)
Supplement: S2 Table — Predictors for fetuin-A concentration (μg/ml) in cerebrospinal fluid. (PDF) [file pone.0268592.s003.pdf]

S2 Table: Multiple linear regression. Predictors for the fetuin-A concentration (µg / ml) in cerebrospinal fluid

**Model summary**	**Adjusted R^2**				
	0.410				
**ANOVA**	**F (2,44)**	**P**			
	17.003	P<0.001			
**Model**	**B***	**Beta****	**Signifi-cance**	**CI for B low**	**CI for B high**
Constant	0.537		0.000	0.362	0.711
Blood-brain barrier dysfunction (no / yes)	1.220	0.736	0.000	0.792	1.648
C-reactive protein (normal / elevated)	-0.889	-0.459	0.001	-1.389	-0.388

* unstandardized coefficients. ** standardized coefficients
