# Supplementary material for: Post-translational modifications glycosylation and phosphorylation of the major hepatic plasma protein fetuin-A are associated with CNS inflammation in children
Source: PLoS One. 2022 Oct 7;17(10):e0268592. doi: 10.1371/journal.pone.0268592 (PMC9544022; doi:10.1371/journal.pone.0268592)
Supplement: S3 Table — Predictors for serum fetuin-A/serum total protein ratio. (PDF) [file pone.0268592.s004.pdf]

S3 Table: Multiple linear regression. Predictors for the serum fetuin-A / serum total protein ratio

**Model summary**	**Adjusted R^2**				
	0.257				
**ANOVA**	**F (2,44)**	**p**			
	8.946	P=0.001			
**Model**	**B***	**Beta****	**Signifi-cance**	**CI for B low**	**CI for B high**
Constant	0.004		P=0.000	0.004	0.005
Blood-brain barrier dysfunction (no / yes)	-0.001	-0.372	P=0.005	-0.001	0.000
Intrathecal IgG synthesis (no / yes)	-0.001	-0.369	P=0.006	-0.001	0.000

* unstandardized coefficients. ** standardized coefficients
