# Supplementary material for: Post-translational modifications glycosylation and phosphorylation of the major hepatic plasma protein fetuin-A are associated with CNS inflammation in children
Source: PLoS One. 2022 Oct 7;17(10):e0268592. doi: 10.1371/journal.pone.0268592 (PMC9544022; doi:10.1371/journal.pone.0268592)
Supplement: S4 Table — Predictors for CSF fetuin-A/CSF total protein ratio. (PDF) [file pone.0268592.s005.pdf]

S4 Table: Multiple linear regression. Predictors for the CSF fetuin-A / CSF total protein ratio

**Model summary**	**Adjusted R^2**				
	0.174				
**ANOVA**	**F (1,45)**	**P**			
	10.715	P=0.002			
**Model**	**B***	**Beta****	**Signifi-cance**	**CI for B low**	**CI for B high**
Constant	0.001		0.077	0.000	0.002
Age (years)	0.0001	0.439	0.002	0.000	0.000

* unstandardized coefficients ** standardized coefficients.

CSF: cerebrospinal fluid.
