# Supplementary material for: Post-translational modifications glycosylation and phosphorylation of the major hepatic plasma protein fetuin-A are associated with CNS inflammation in children
Source: PLoS One. 2022 Oct 7;17(10):e0268592. doi: 10.1371/journal.pone.0268592 (PMC9544022; doi:10.1371/journal.pone.0268592)
Supplement: S5 Table — Predictors for the CSF fetuin-A/serum fetuin-A quotient. (PDF) [file pone.0268592.s006.pdf]

S5 Table: Multiple linear regression. Predictors for the CSF fetuin-A / serum fetuin-A ratio

**Model summary**	**Adjusted R^2**				
	0.878				
**ANOVA**	**ΔF (4,42)**	**p**			
	83.860	P<0.001			
**Model**	**B***	**Beta****	**Signifi-cance**	**CI for B low**	**CI for B high**
Constant	-0.020		0.957	-0.782	0.741
albumin CSF/serum ratio	0.191	0.761	0.000	0.159	0.223
Age (years)	0.077	0.150	0.007	0.022	0.132
Blood-brain barrier disruption (no / yes)	1.548	0.264	0.001	0.704	2.391
C-reactive protein (normal / elevated)	-1.151	-0.168	0.010	-2.016	-0.286

* unstandardized coefficients; ** standardized coefficients
