# Supplementary material for: Post-translational modifications glycosylation and phosphorylation of the major hepatic plasma protein fetuin-A are associated with CNS inflammation in children
Source: PLoS One. 2022 Oct 7;17(10):e0268592. doi: 10.1371/journal.pone.0268592 (PMC9544022; doi:10.1371/journal.pone.0268592)
Supplement: S6 Table — Predictors for relative CSF phosphofetuin-A concentrations. (PDF) [file pone.0268592.s007.pdf]

S6 Table: Multiple linear regression. Predictors for relative CSF phosphofetuin-A concentrations.

**Model summary**	**Adjusted R^2**				
	0.506				
**ANOVA**	**F (2,27)**	**P**			
	15.860	P<0.001			
**Model**	**B***	**Beta****	**Signifi-cance**	**CI for B low**	**CI for B high**
Constant	4.555		0.016	0.911	8.198
CSF / serum albumin ratio	0.396	0.709	0.000	0.244	0.547
Not inflammatory / inflammatory	-5.262	-0.342	0.016	-9.442	-1.083

* unstandardized coefficients; ** standardized coefficients
