# Supplementary material for: Post-translational modifications glycosylation and phosphorylation of the major hepatic plasma protein fetuin-A are associated with CNS inflammation in children
Source: PLoS One. 2022 Oct 7;17(10):e0268592. doi: 10.1371/journal.pone.0268592 (PMC9544022; doi:10.1371/journal.pone.0268592)
Supplement: S7 Table — Predictors for absolute CSF phosphofetuin-A concentrations. (PDF) [file pone.0268592.s008.pdf]

S7 Table: Multiple linear regression. Predictors for relative CSF phosphofetuin-A concentrations.

**Model summary**	**Adjusted R^2**				
	0.907				
**ANOVA**	**F (2,27)**	**P**			
	143.039	P<0.001			
**Model**	**B***	**Beta****	**Signifi-cance**	**CI for B low**	**CI for B high**
Constant	-0.027		0.001	-0.042	-0.012
CSF albumin / serum albumin ratio	0.010	1,065	0.000	0.009	0.011
Blood brain barrier	-0.060	-0.222	0.003	-0.099	-0.022

* unstandardized coefficients; ** standardized coefficients
