# Supplementary material for: Post-translational modifications glycosylation and phosphorylation of the major hepatic plasma protein fetuin-A are associated with CNS inflammation in children
Source: PLoS One. 2022 Oct 7;17(10):e0268592. doi: 10.1371/journal.pone.0268592 (PMC9544022; doi:10.1371/journal.pone.0268592)
Supplement: S8 Table — Predictors for relative CSF phosphofetuin-A/relative serum phosphofetuin-A quotient. (PDF) [file pone.0268592.s009.pdf]

S8 Table: Multiple linear regression. Predictors for the ratio relative CSF phosphofetuin-A / relative serum phosphofetuin-A levels.

**Model summary**	**Adjusted R^2**				
	0.521				
**ANOVA**	**F (2,26)**	**P**			
	16.220	P<0.001			
**Model**	**B***	**Beta****	**Signifi-cance**	**CI for B low**	**CI for B high**
Constant	0.751		0.010	0.196	1.305
CSF / serum albumin ratio	0.061	0.717	0.000	0.038	0.084
Not inflammatory / inflammatory	-0.821	-0.351	0.014	-1.457	-0.184

* unstandardized coefficients; ** standardized coefficients
