# Supplementary material for: Post-translational modifications glycosylation and phosphorylation of the major hepatic plasma protein fetuin-A are associated with CNS inflammation in children
Source: PLoS One. 2022 Oct 7;17(10):e0268592. doi: 10.1371/journal.pone.0268592 (PMC9544022; doi:10.1371/journal.pone.0268592)
Supplement: S1 Raw images — (PDF) [file pone.0268592.s010.pdf]

glycosylation studies

Lanes	1	2	3	4	5	6	7	8	9	10
**Gel:**										
S1 glyco	Marker	S1 no	S1 N	S1 S	S3 no	S3 N	S3 S	S7 no	S7 N	S7 S
S2 glyco	Marker	S9 no	S9 N	S9 S	S12 no	S12 N	S12 S	S22 no	S22 N	S22 S
S3 glyco	Marker	S25 no	S25 N	S25 S	S32 no	S32 N	S32 S	S33 no	S33 N	S33 S
S4 glyco	Marker	S35 no	S35 N	S35 S	S41 no	S41 N	S41 S	S50 no	S50 N	S50 S
S5 glyco	Marker	S40 no	S40 N	S40 S	S45 no	S45 N	S45 S	S47 no	S47 N	S47 S
S6 glyco	Marker	S55 no	S55 N	S55 S	S57 no	S57 N	S57 S	S58 no	S58 N	S58 S
S7 glyco	Marker	S61 no	S61 N	S61 S	S62 no	S62 N	S62 S	S63 no	S63 N	S63 S
S8 glyco	Marker	S66 no	S66 N	S66 S	S69 no	S69 N	S69 S			
CSF1 glyco	Marker	CSF1 no	CSF1 N	CSF1 S	CSF3 no	CSF3 N	CSF3 S	CSF7 no	CSF7 N	CSF7 S
CSF2 glyco	Marker				CSF12 N	CSF12 no	CSF12 S	CSF22 no	CSF22 N	CSF22 S
CSF3 glyco	Marker	CSF25 no	CSF25 N	CSF25 S	CSF32 no	CSF32 N	CSF32 S	CSF33 no	CSF33 N	CSF33 S
CSF4 glyco	Marker	CSF35 no	CSF35 N	CSF35 S	CSF41 no	CSF41 N	CSF41 S	CSF50 no	CSF50 N	CSF50 S
CSF5 glyco	Marker	CSF40 no	CSF40 N	CSF40 S	CSF45 no	CSF45 N	CSF45 S	CSF47 no	CSF47 N	CSF47 S
CSF6 glyco	Marker	CSF55 no	CSF55 N	CSF55 S	CSF57 no	CSF57 N	CSF57 S			
CSF7 glyco	Marker	CSF61 no	CSF61 N	CSF61 S	CSF62 no	CSF62 N	CSF62 S	CSF63 no	CSF63 N	CSF63 S
CSF8 glyco	Marker	CSF66 no	CSF66 N	CSF66 S	CSF69 no	CSF69 N	CSF69 S			
CSF9 glyco	Marker	CSF9 no	CSF9 N	CSF9 S	CSF50 no	CSF50 N	CSF50 S	CSF58 no	CSF58 N	CSF58 S

no = no digestion

N = digestion with PNGase-F

S=digestion with sialidase-Au

PNGase kinetics

	1	2	3	4	5	6	7	8	9	10
S_CSF PNG	S a no	S a N 1 min	S a N 10 min	S a N 3 h	CSF a no	CSF a N 1 min	CSF a N 10 mi	CSF a N 3 h		
CSF PNG	CSF b no	CSF b N 1 min	CSF b N 10 min	CSF b N 3 h	CSF b no	CSF b O 1 min	CSF b O 10 mi	CSF b O 3 h		

N = digestion with PNGase-F

O = digestion with sialidase-Au + O Glykosidase

Used for figure 2:

Fig 2 A:

	gel	*lanes*
ser 1:	S3 glyco	2, 3, 4
CSF 1:	CSF3 glyco	2, 3, 4
ser 2:	S1 glyco	8, 9, 10
CSF 2:	CSF1 glyco	8, 9, 10

Fig 2 B:

	gel	*lanes*
ser 3:	S3 glyco	2, 3, 4
CSF 3:	CSF 3 glyco	2, 3, 4
ser 4:	S3 glyco	5, 6, 7
CSF 4:	CSF 3 glyco	5, 6, 7

Fig 2 D:

	gel	lanes
ser	S_CSF PNG	1, 2, 3, 4
CSF	CSF PNG	1, 2, 3, 4

Fig 2 C:

statistics from all glycosylation blots (S1 glyco - S8 glyco, CSF1 glyco - CSF9 glyco)

Phosphorylation studies

lanes	1	2	3	4	5	6	7	8	9	10	11	12	13	14	15	16	17
S1 phosph	S31 P	M	S30	S30 P	S17	S17 P	S26	S26 P	S16	S16 P	S56	S56 P	S27	S27 P	S53	S53 P	S31
SCSF1 phosph	M	S11	S11 P	S36	S36 P	S65	S65 P	S2	S2 P	CSF 11	CSF11 P	CSF36	CSF36 P	CSF65	CSF65 P	CSF2	CSF2 P
S2 phosph	M	S4	S4 P	S60	S60 P	S37	S37 P	S42	S42 P	S10	S10 P	S6	S6 P	S51	S51 P	S70	S70 P
S3 phosph	M	S1	S1 P	S3	S3 P	S7	S7 P	S9	S9 P	S12	S12 P	S22	S22 P	S25	S25 P	S32	S32 P
S4 phosph	M	S33	S33 P	S35	S35 P	S40	S40 P	S41	S41 P	S45	S45 P	S47	S47 P	S50	S50 P	S55	S55 P
S5 phosph	M	S57	S57 P	S61	S61 P	S62	S62 P	S63	S63 P	S66	S66 P	S69	S69 P				
S6 phosph	S62	S62 P	S55	S55 P	S1	S1 P	S33	S33 P	STA	S32	S32 P	S47	S47 P	S50	S50 P	S25	S25 P
S7 phosph	S3	S3 P	S40	S40 P	S63	S63P	S57	S57 P	STA	S41	S41 P	S35	S35 P	S69	S69 P	S7	S7 P
S8 phosph	S12	S12P	S66	S66 P	S22	S22 P	S45	S45 P	STA	S61	S61 P						
CSF1 phosph	M	CSF30	CSF30 P	CSF17	CSF17 P	CSF26	CSF26 P	CSF16	CSF16 P	CSF56	CSF56 P	CSF27	CSF27 P	CSF53	CSF53 P	CSF31	CSF31 P
CSF2 phosph	M	CSF4	CSF4P	CSF60	CSF60 P	CSF37	CSF37 P	CSF42	CSF42 P	CSF10	CSF10 P	CSF6	CSF6 P	CSF51	CSF51 P	CSF70	CSF70 P
CSF3 phosph	M	CSF1	CSF1 P	CSF3	CSF3 P	CSF7	CSF7 P	CSF9*	CSF9 P*	CSF12	CSF12 P	CSF22	CSF22 P	CSF25	CSF25 P	CSF32	CSF32 P
CSF4 phosph	M	CSF33	CSF33 P	CSF35	CSF35 P	CSF40	CSF40 P	CSF41	CSF41 P	CSF45	CSF45 P	CSF47	CSF47 P	CSF50	CSF50 P	CSF55	CSF55 P
CSF5 phosph	CSF62	CSF62 P	CSF55	CSF55 P	CSF1	CSF1 P	CSF33	CSF33 P	STA	CSF32	CSF32 P	CSF47	CSF47 P	CSF50	CSF50 P	CSF25	CSF25 P
CSF6 phosph	CSF3	CSF3 P	CSF40	CSF40 P	CSF63	CSF63 P	CSF57	CSF57 P	STA	CSF41	CSF41 P	CSF35	CSF35 P	CSF69	CSF69 P	CSF7	CSF7 P
CSF7 phosph	CSF12	CSF12 P	CSF66	CSF66 P	CSF22	CSF22 P	CSF45	CSF45 P	STA	CSF61	CSF61 P						
CSF8 phosph	M	CSF57	CSF57 P	CSF61	CSF61 P	CSF62	CSF62 P	CSF63	CSF63 P	CSF66	CSF66 P	CSF69	CSF69 P				

P = digestion with alkaline phosphatase * not measurable

STA= = internal standard (S3)

all gels used for statistical analysis for Fig 3 (CSF 9 was not measurable, therefore CSF 9 and S 9 were not used for phosphofetuin analysis)

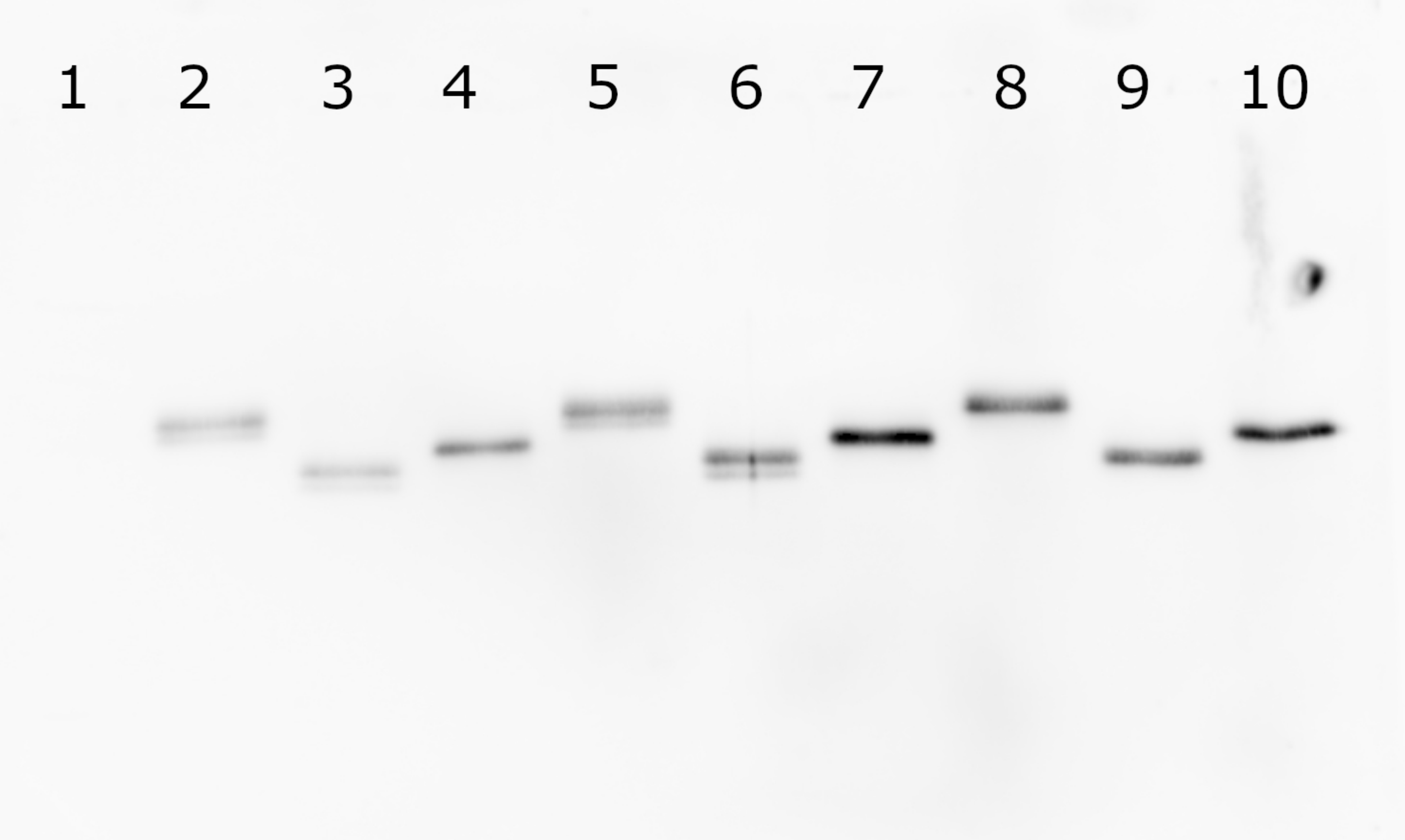

1	2	3	4	5	6	7	8	9	10
Marker	S1 no	S1 N	S1 S	S3 no	S3 N	S3 S	S7 no	S7 N	S7 S

S1 glyco

1	2	3	4	5	6	7	8	9	10
Marker	S9 no	S9 N	S9 S	S12 no	S12 N	S12 S	S22 no	S22 N	S22 S

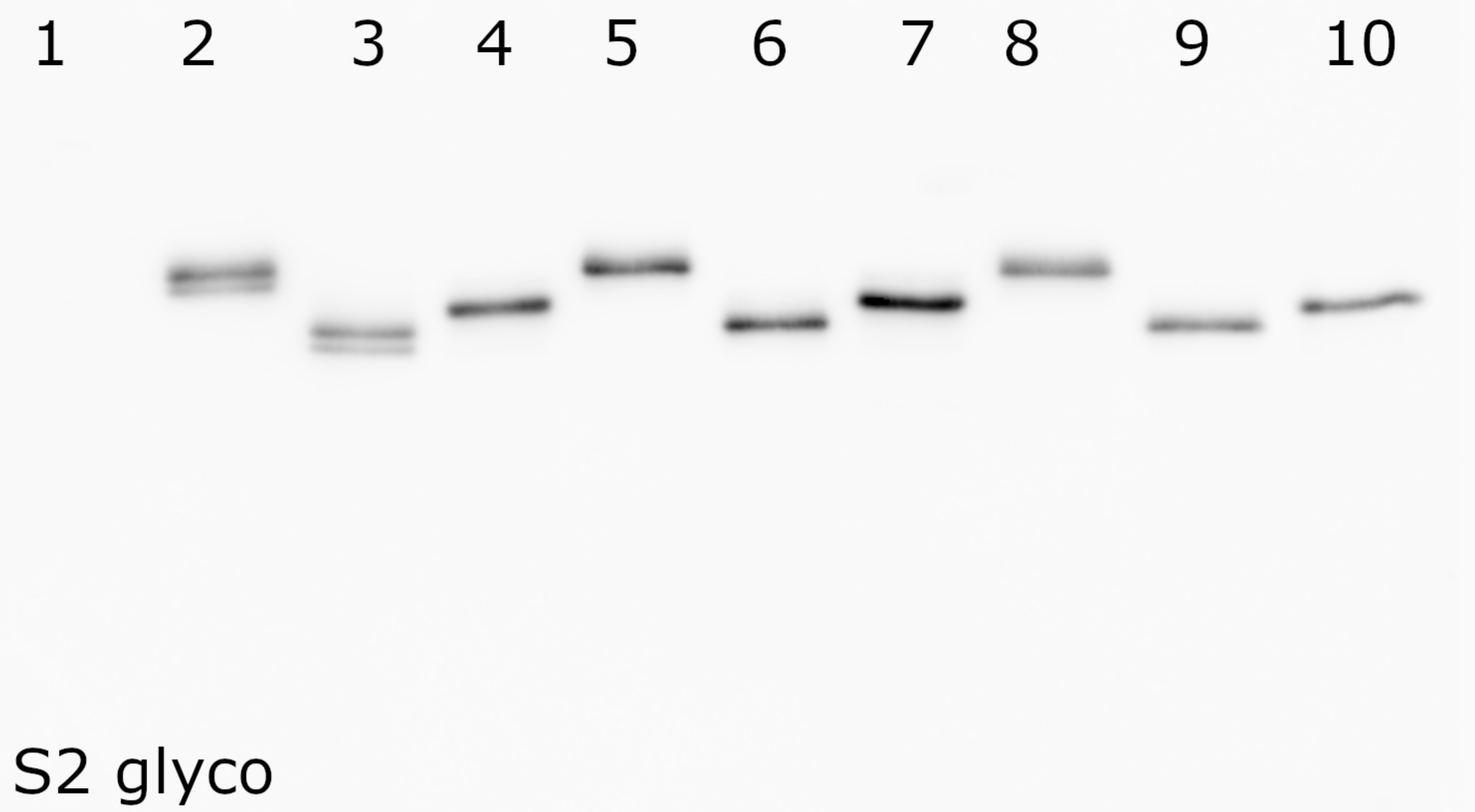

1	2	3	4	5	6	7	8	9	10
Marker	S25 no	S25 N	S25 S	S32 no	S32 N	S32 S	S33 no	S33 N	S33 S

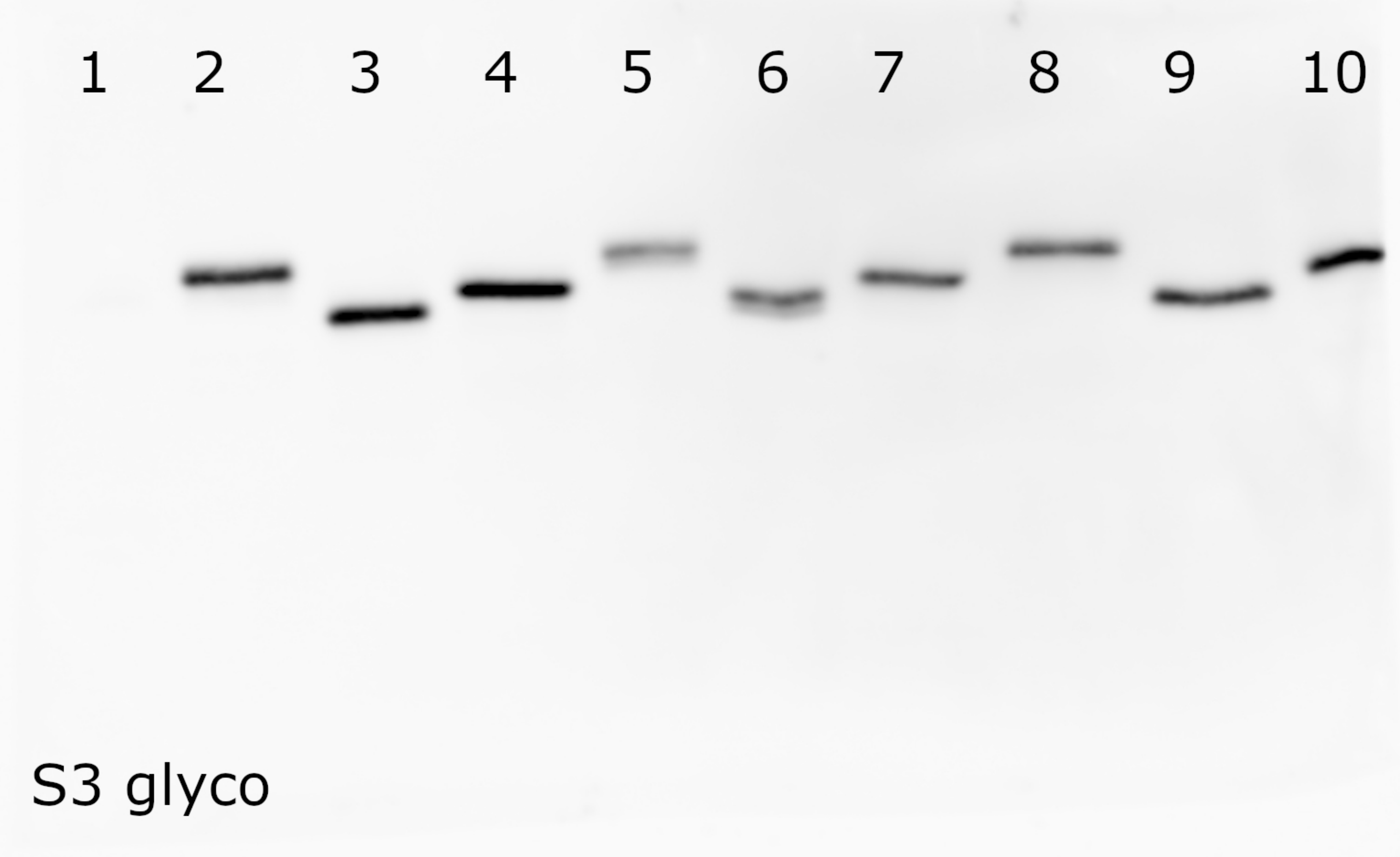

1	2	3	4	5	6	7	8	9	10
Marker	S35 no	S35 N	S35 S	S41 no	S41 N	S41 S	S50 no	S50 N	S50 S

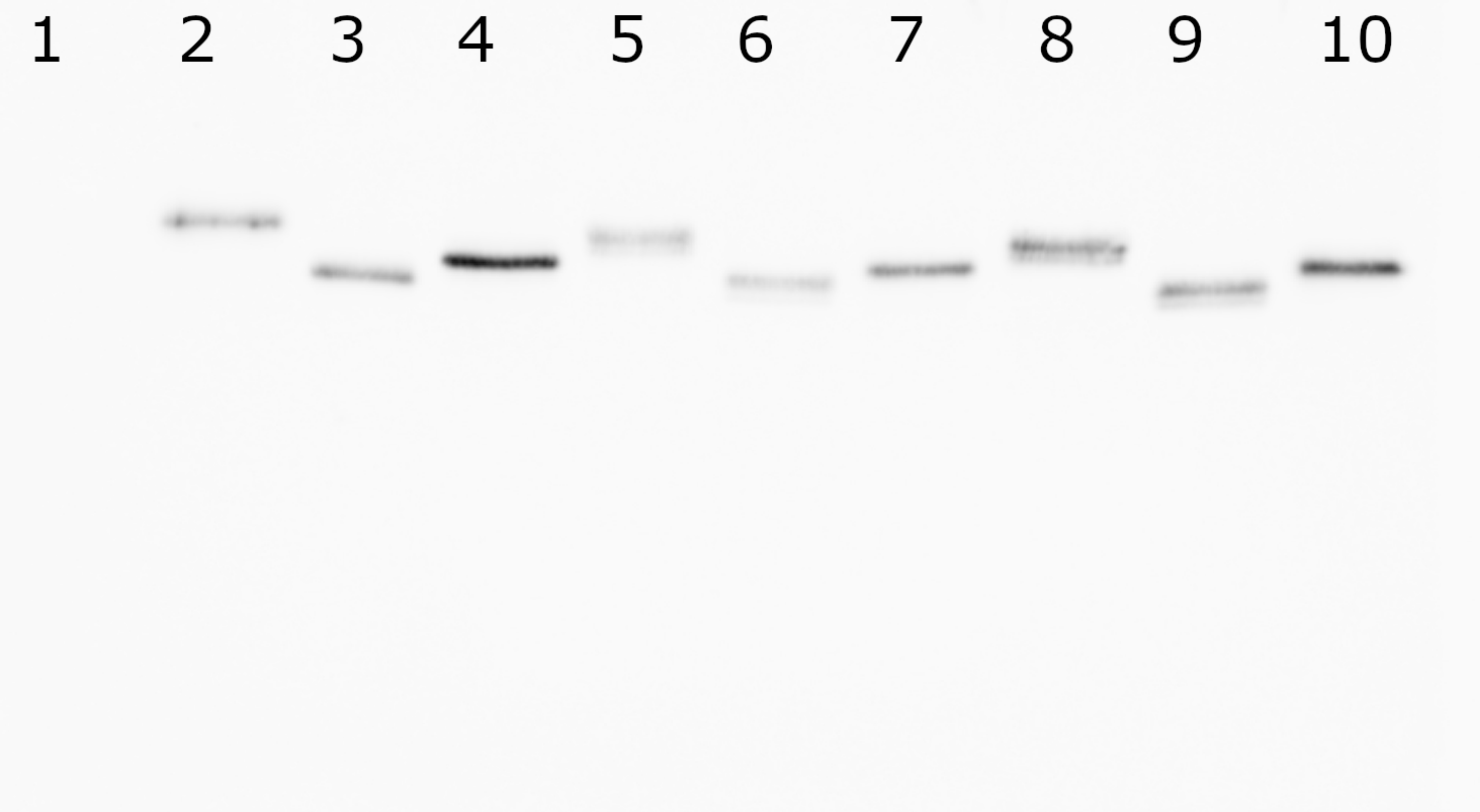

S4 glyco

1	2	3	4	5	6	7	8	9	10
Marker	S40 no	S40 N	S40 S	S45 no	S45 N	S45 S	S47 no	S47 N	S47 S

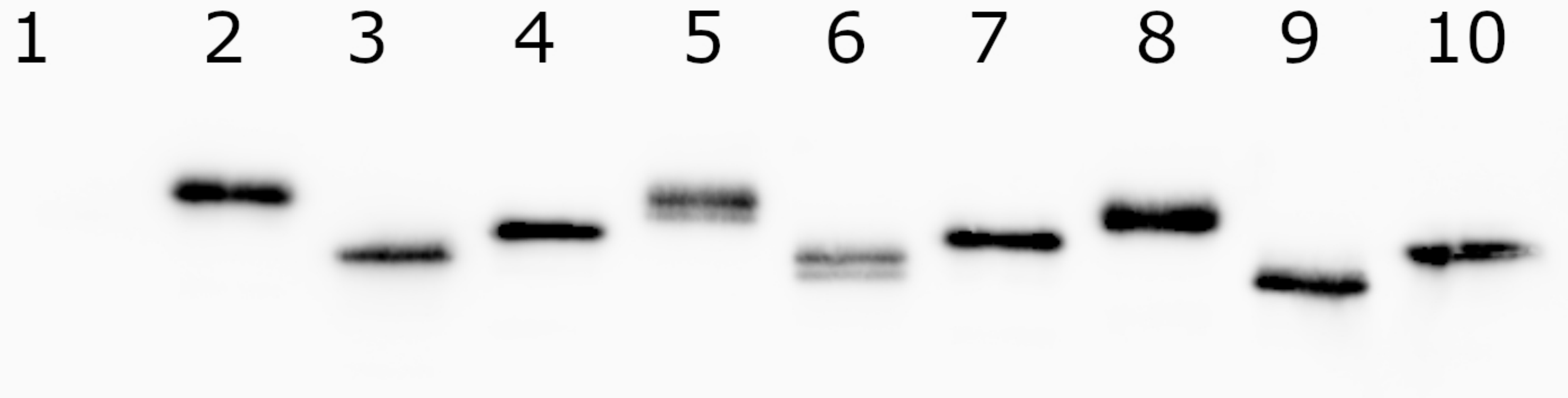

S5 glyco

1	2	3	4	5	6	7	8	9	10
Marker	S55 no	S55 N	S55 S	S57 no	S57 N	S57 S	S58 no	S58 N	S58 S

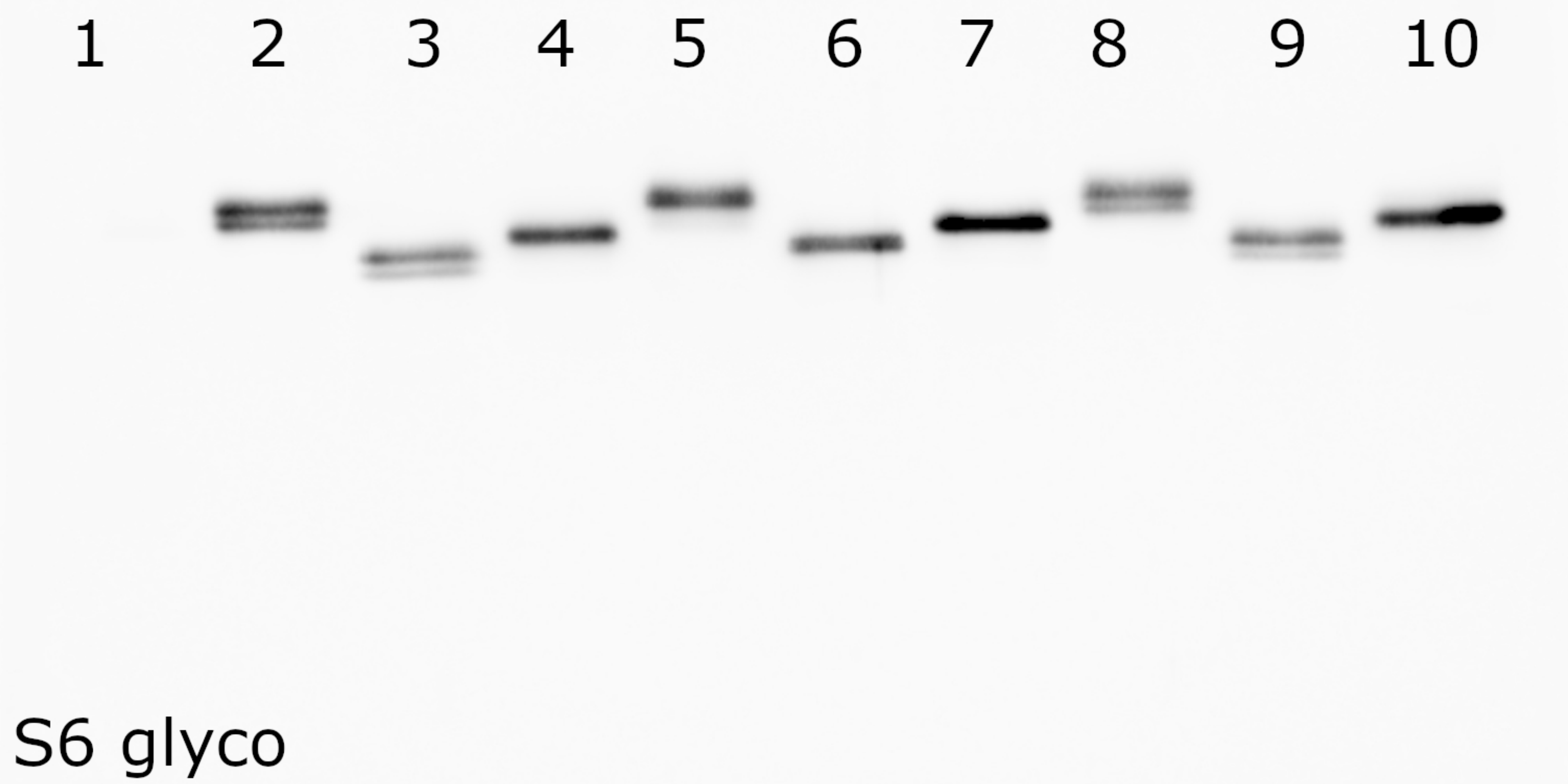

1	2	3	4	5	6	7	8	9	10
Marker	S61 no	S61 N	S61 S	S62 no	S62 N	S62 S	S63 no	S63 N	S63 S

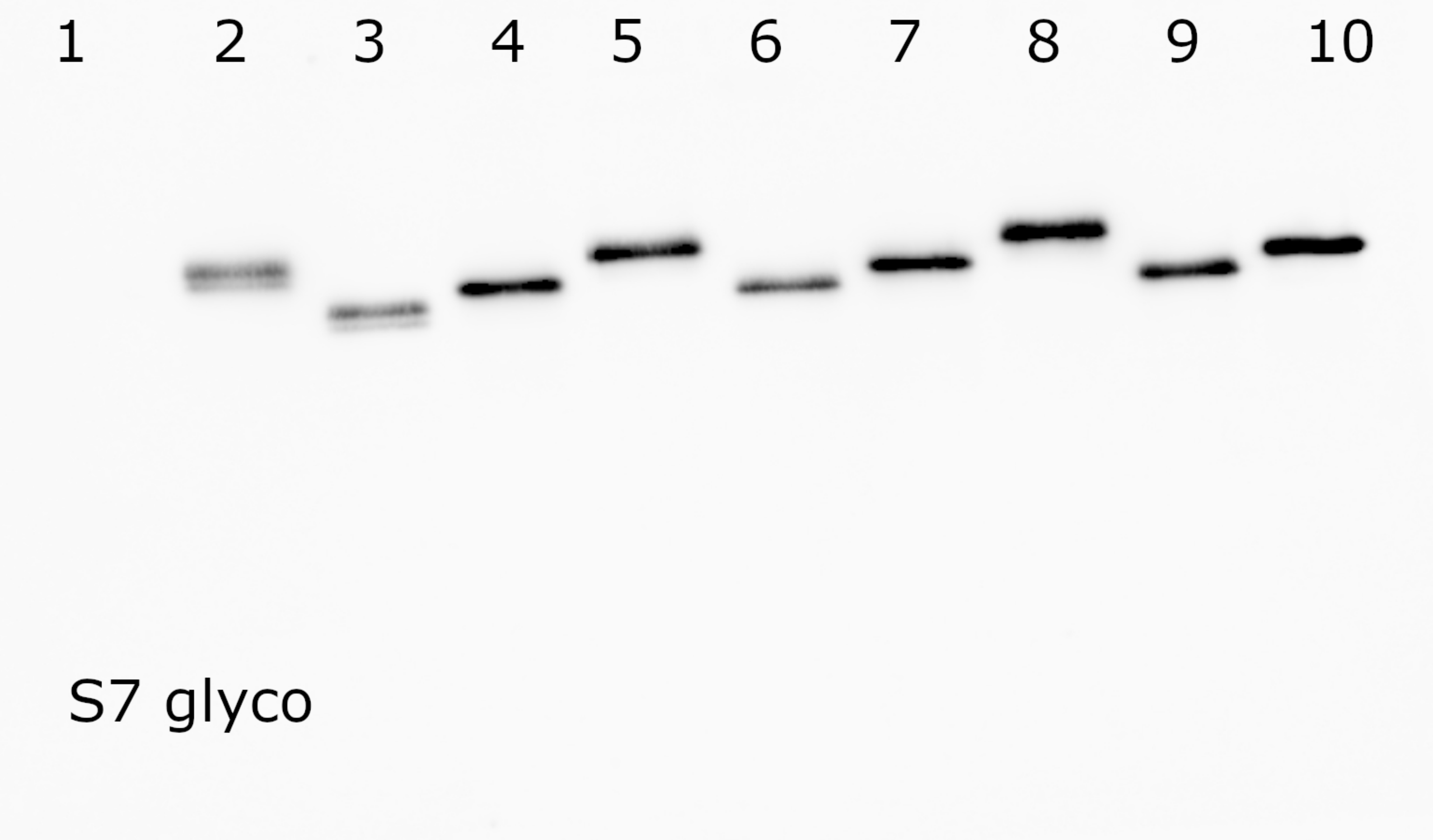

1	2	3	4	5	6	7	8	9	10
Marker	S66 no	S66 N	S66 S	S69 no	S69 N	S69 S			

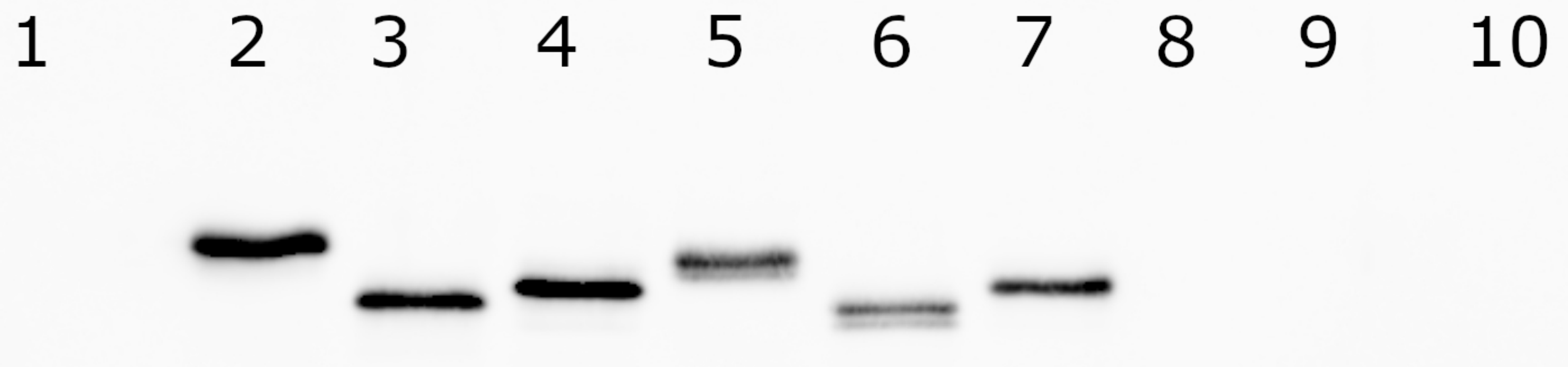

S8 glyco

1	2	3	4	5	6	7	8	9	10
Marker	CSF1 no	CSF1 N	CSF1 S	CSF3 no	CSF3 N	CSF3 S	CSF7 no	CSF7 N	CSF7 S

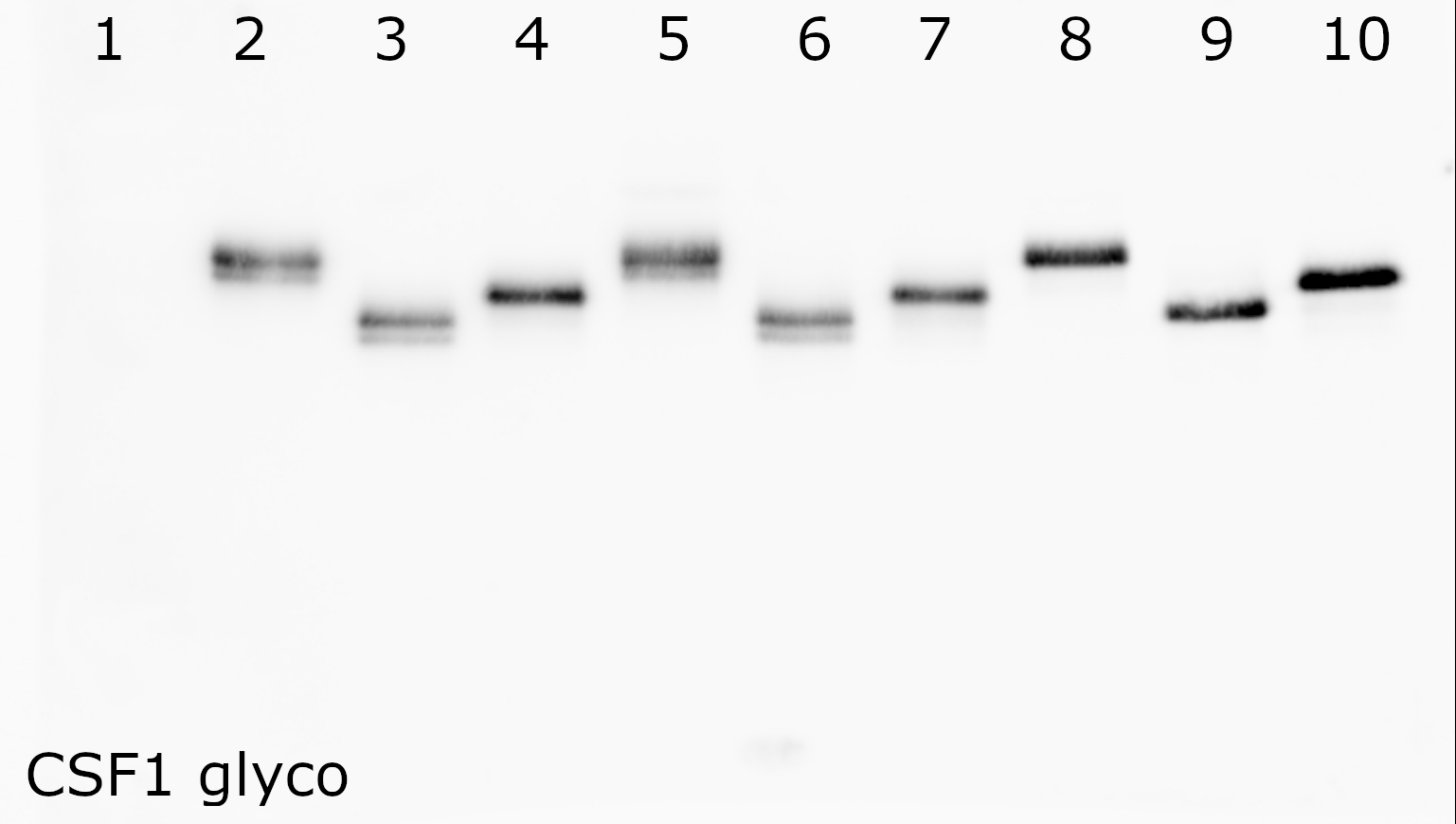

1	2	3	4	5	6	7	8	9	10
Marker				CSF12 N	CSF12 no	CSF12 S	CSF22 no	CSF22 N	CSF22 S

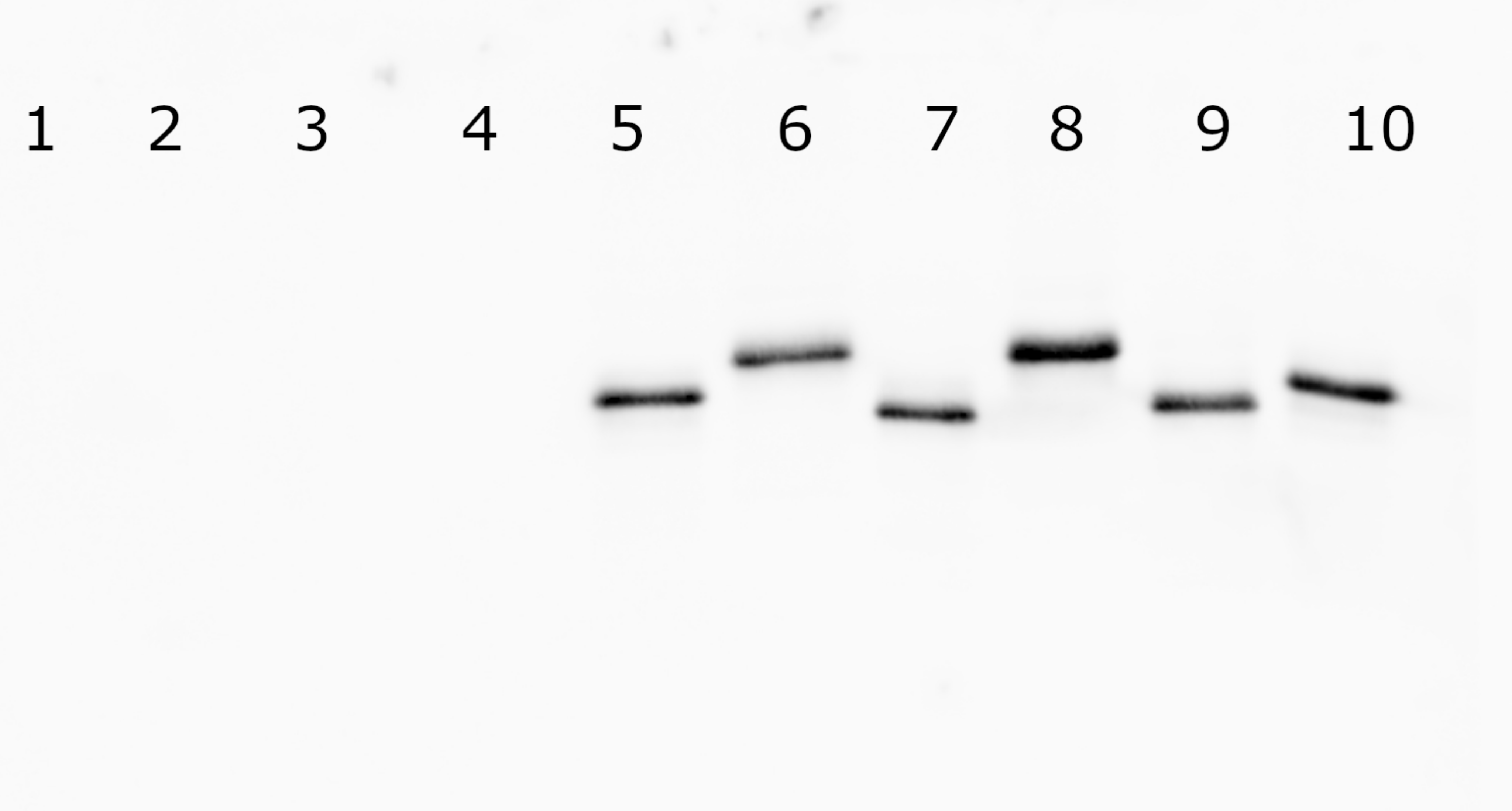

CSF2 glyco

1	2	3	4	5	6	7	8	9	10
Marker	CSF25 no	CSF25 N	CSF25 S	CSF32 no	CSF32 N	CSF32 S	CSF33 no	CSF33 N	CSF33 S

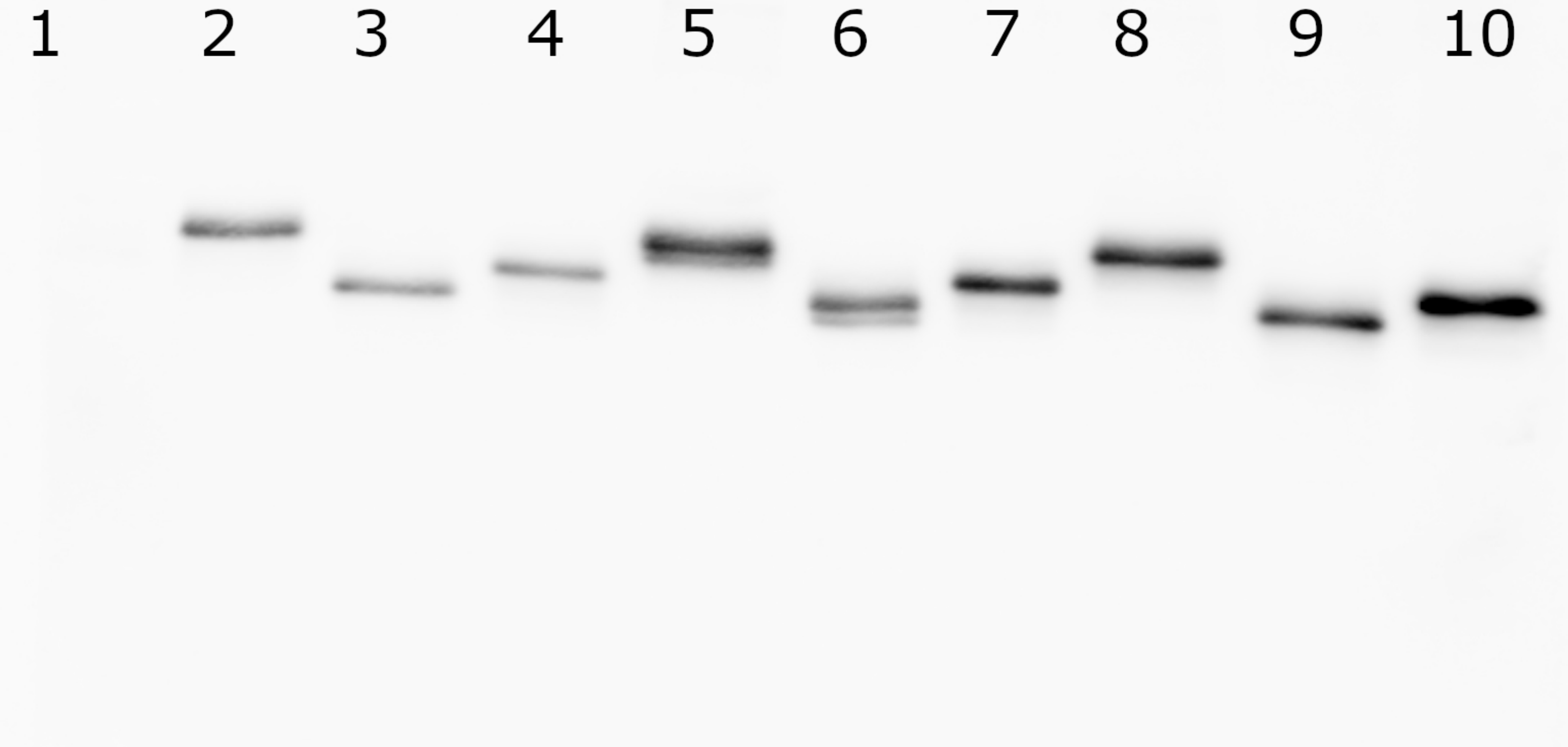

CSF3 glyco

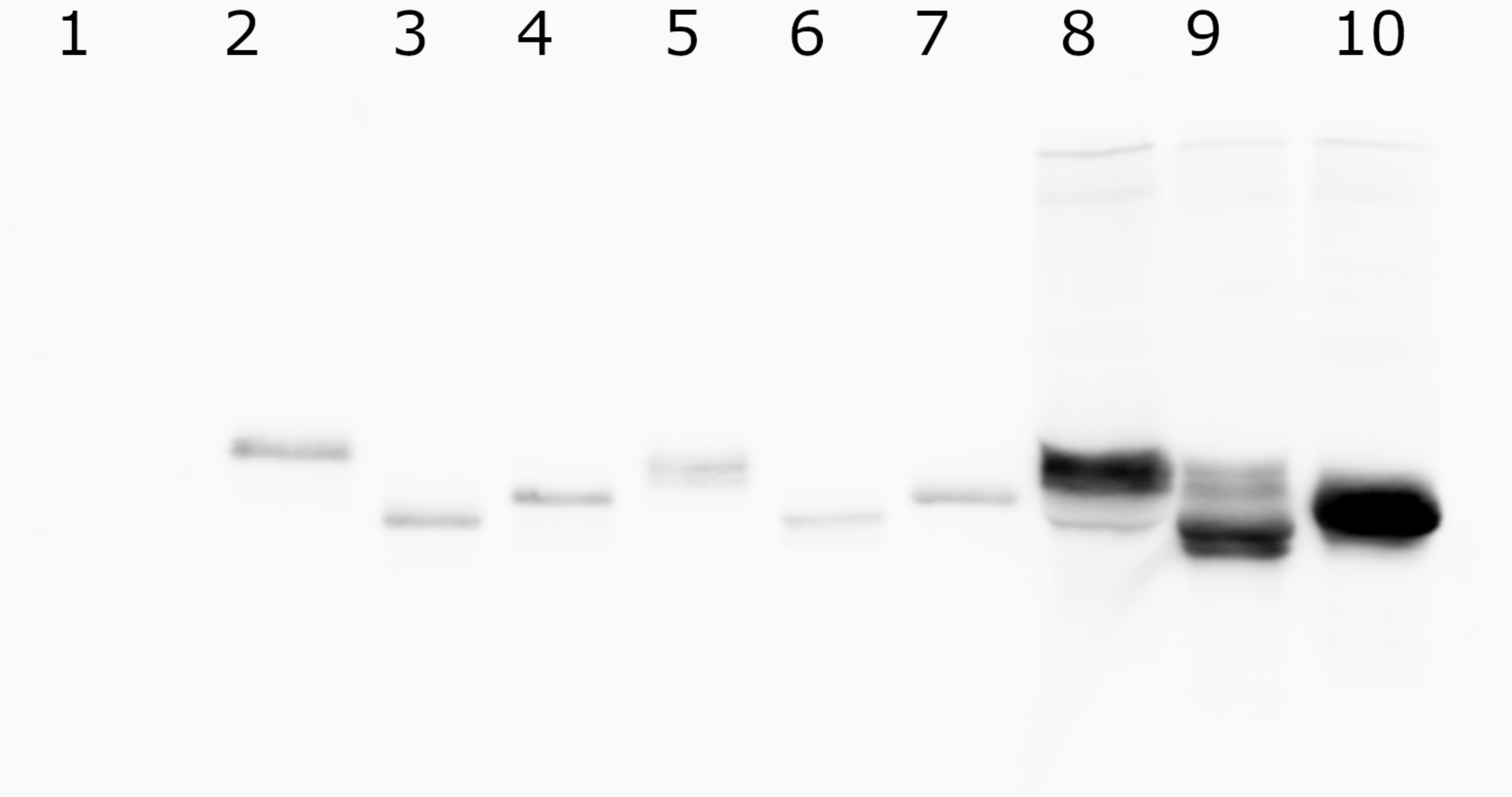

CSF4 glyco

1	2	3	4	5	6	7	8	9	10
Marker	CSF35 no	CSF35 N	CSF35 S	CSF41 no	CSF41 N	CSF41 S	CSF50 no	CSF50 N	CSF50 S

1	2	3	4	5	6	7	8	9	10
Marker	CSF40 no	CSF40 N	CSF40 S	CSF45 no	CSF45 N	CSF45 S	CSF47 no	CSF47 N	CSF47 S

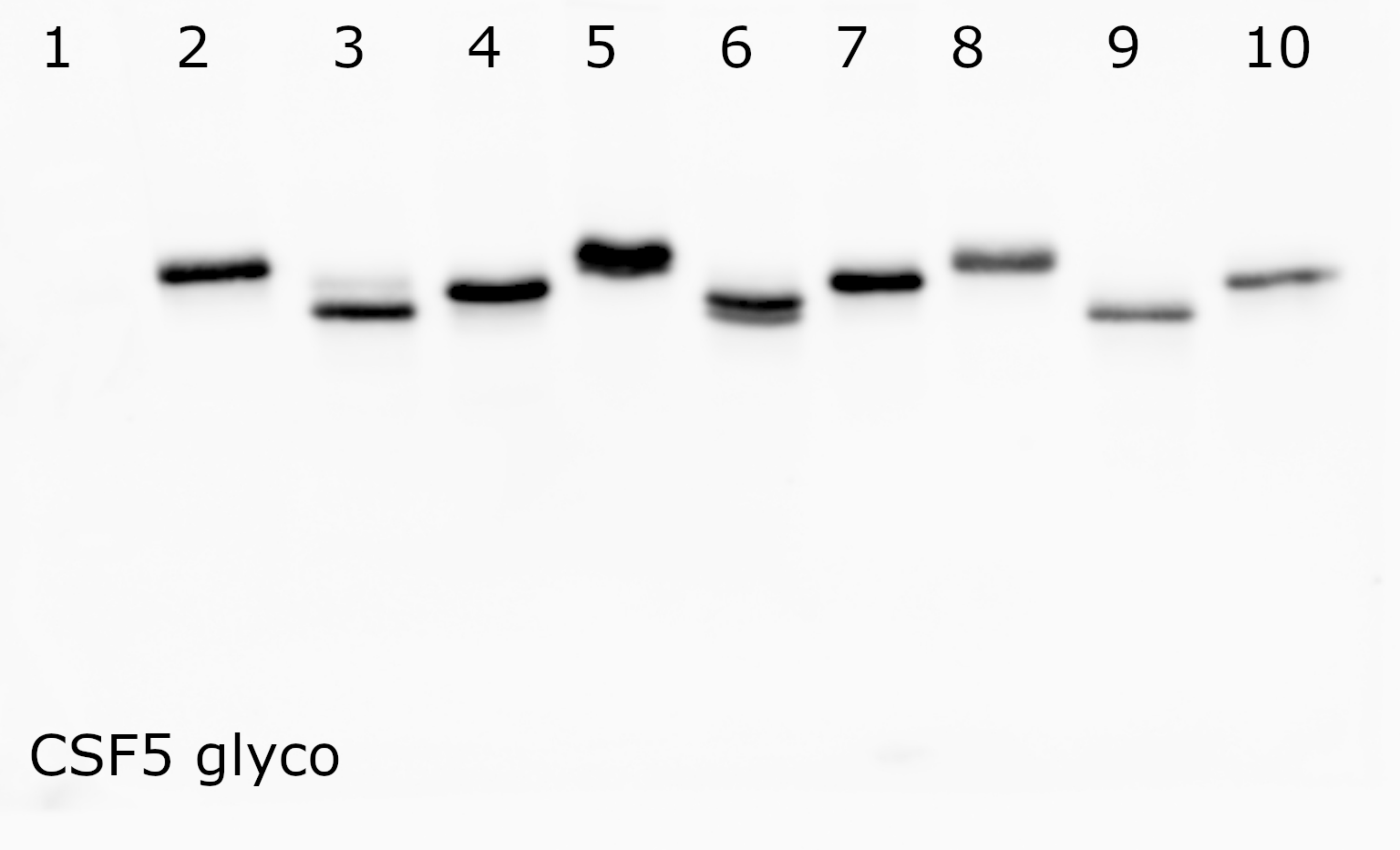

1	2	3	4	5	6	7	8	9	10
Marker	CSF55 no	CSF55 N	CSF55 S	CSF57 no	CSF57 N	CSF57 S			

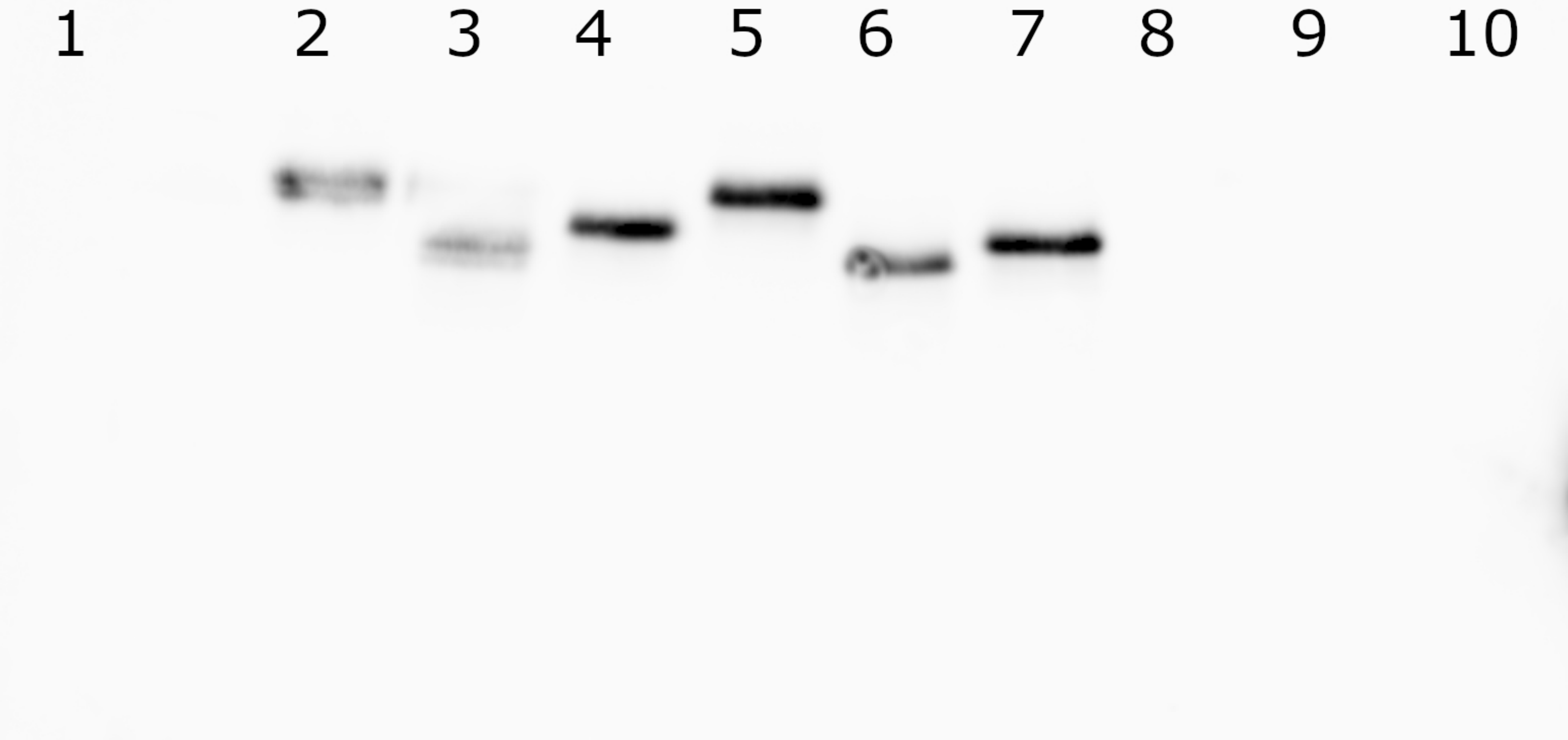

CSF6 glyco

1	2	3	4	5	6	7	8	9	10
Marker	CSF61 no	CSF61 N	CSF61 S	CSF62 no	CSF62 N	CSF62 S	CSF63 no	CSF63 N	CSF63 S

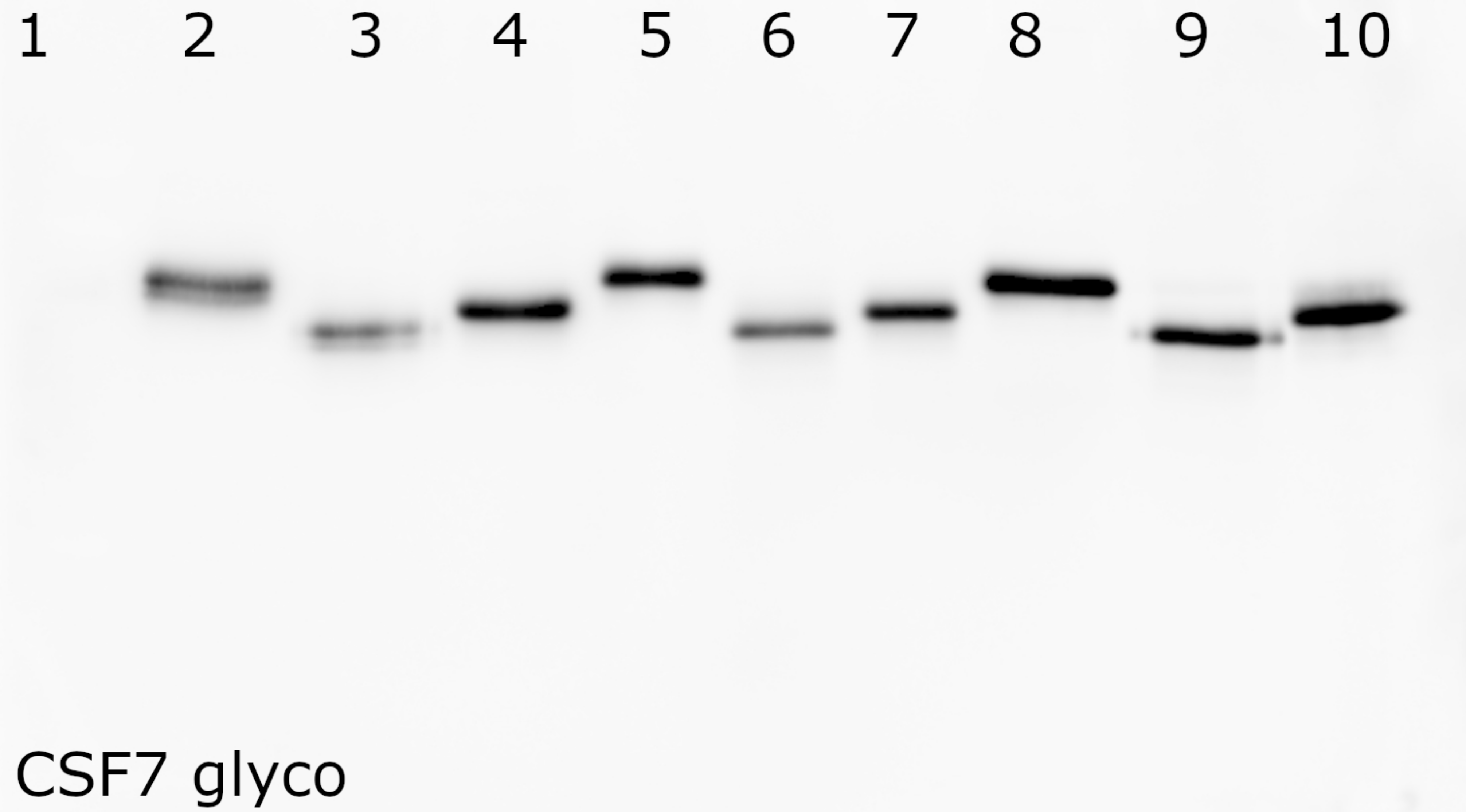

1	2	3	4	5	6	7	8	9	10
Marker	CSF66 no	CSF66 N	CSF66 S	CSF69 no	CSF69 N	CSF69 S			

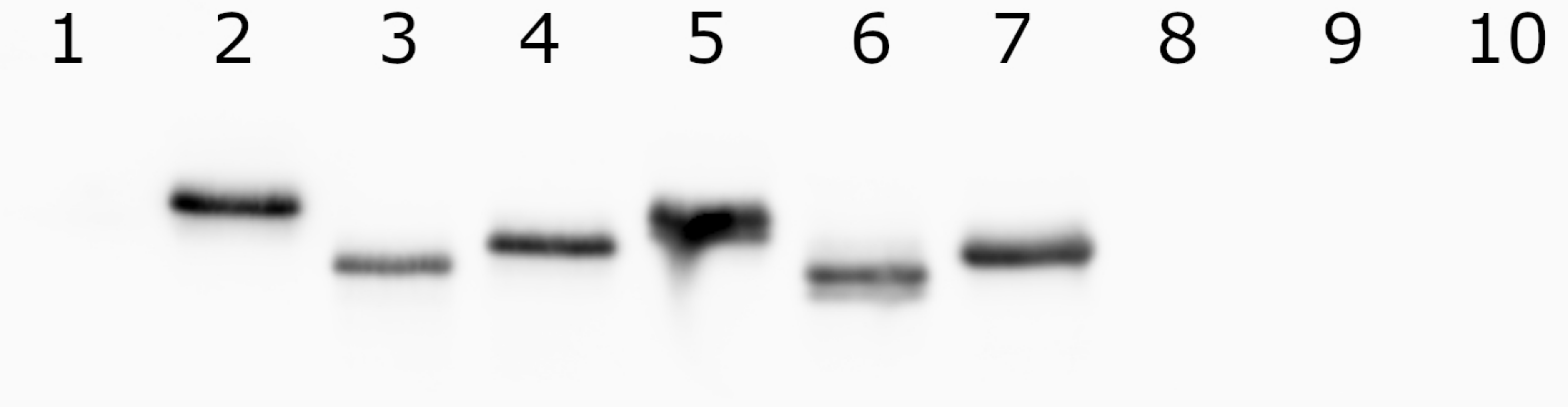

CSF 8 glyco

1	2	3	4	5	6	7	8	9	10
Marker	CSF9 no	CSF9 N	CSF9 S	CSF50 no	CSF50 N	CSF50 S	CSF58 no	CSF58 N	CSF58 S

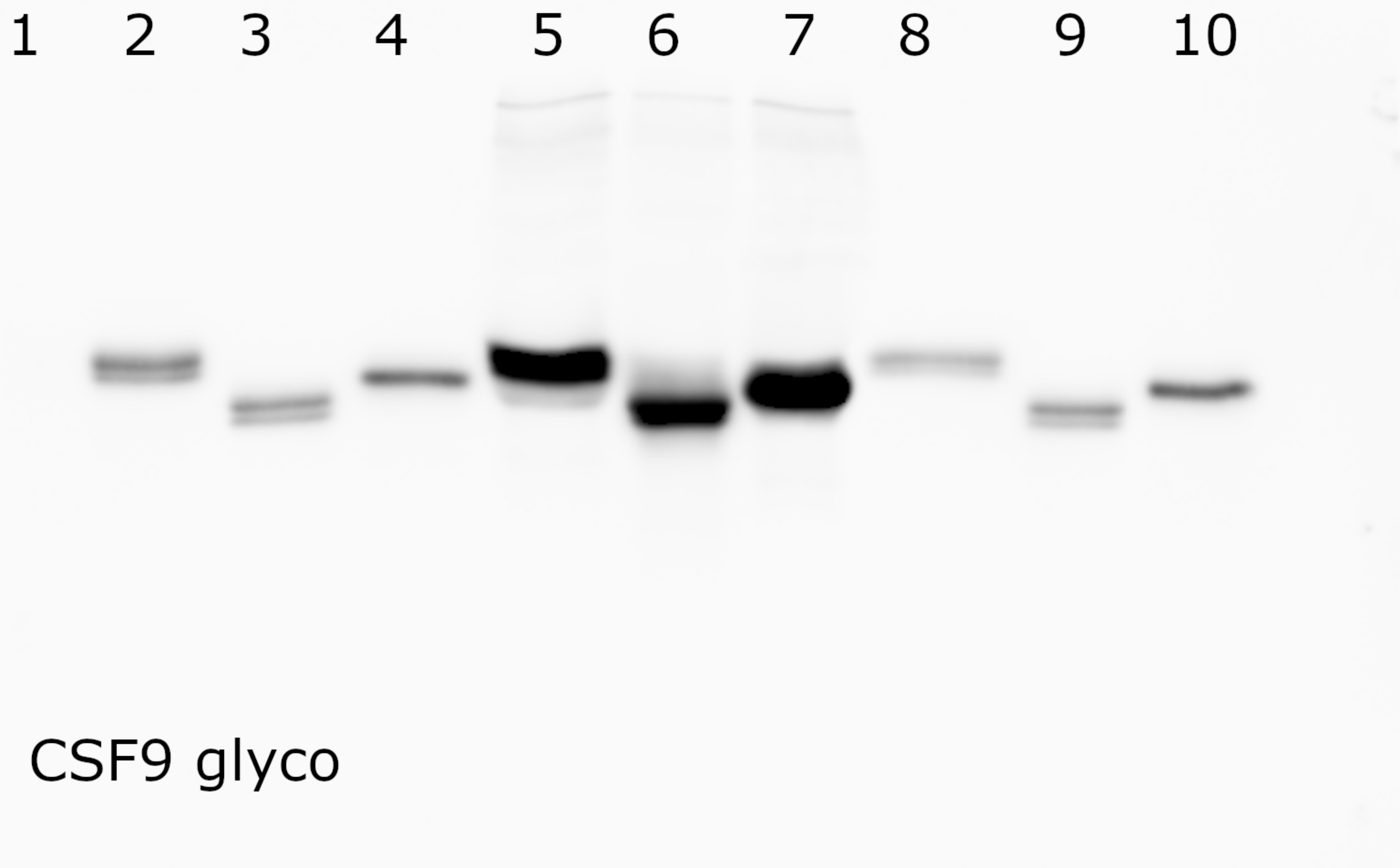

1	2	3	4	5	6	7	8
S a no	S a N 1 min	S a N 10 min	S a N 3 h	CSF a no	CSF a N 1 min	CSF a N 10 min	CSF a N 3 h

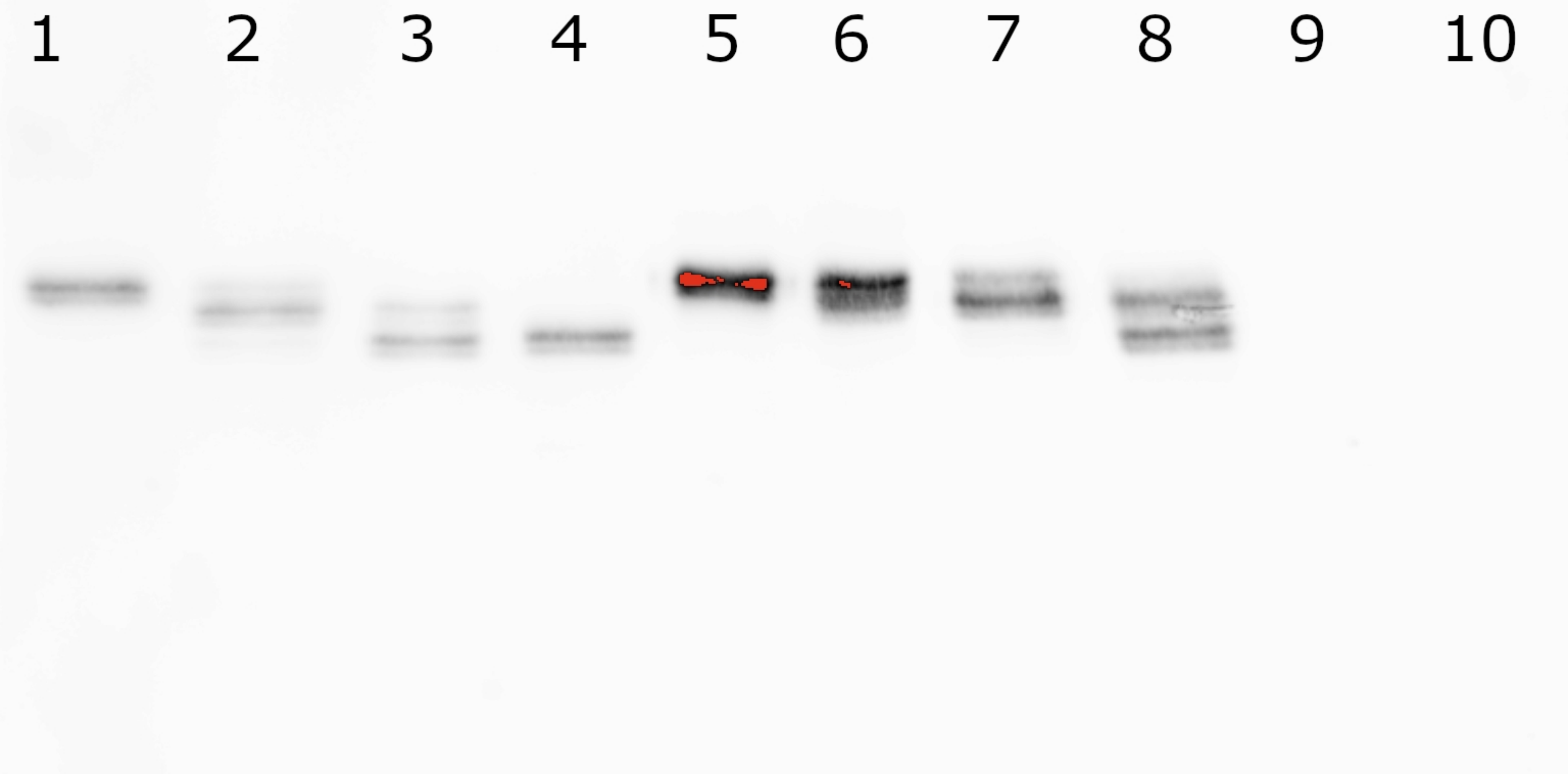

S_CSF PNG

1	2	3	4	5	6	7	8
CSF b no	CSF b N 1 min	CSF b N 10 min	CSF b N 3 h	CSF b no	CSF b O 1 min	CSF b O 10 min	CSF b O 3 h

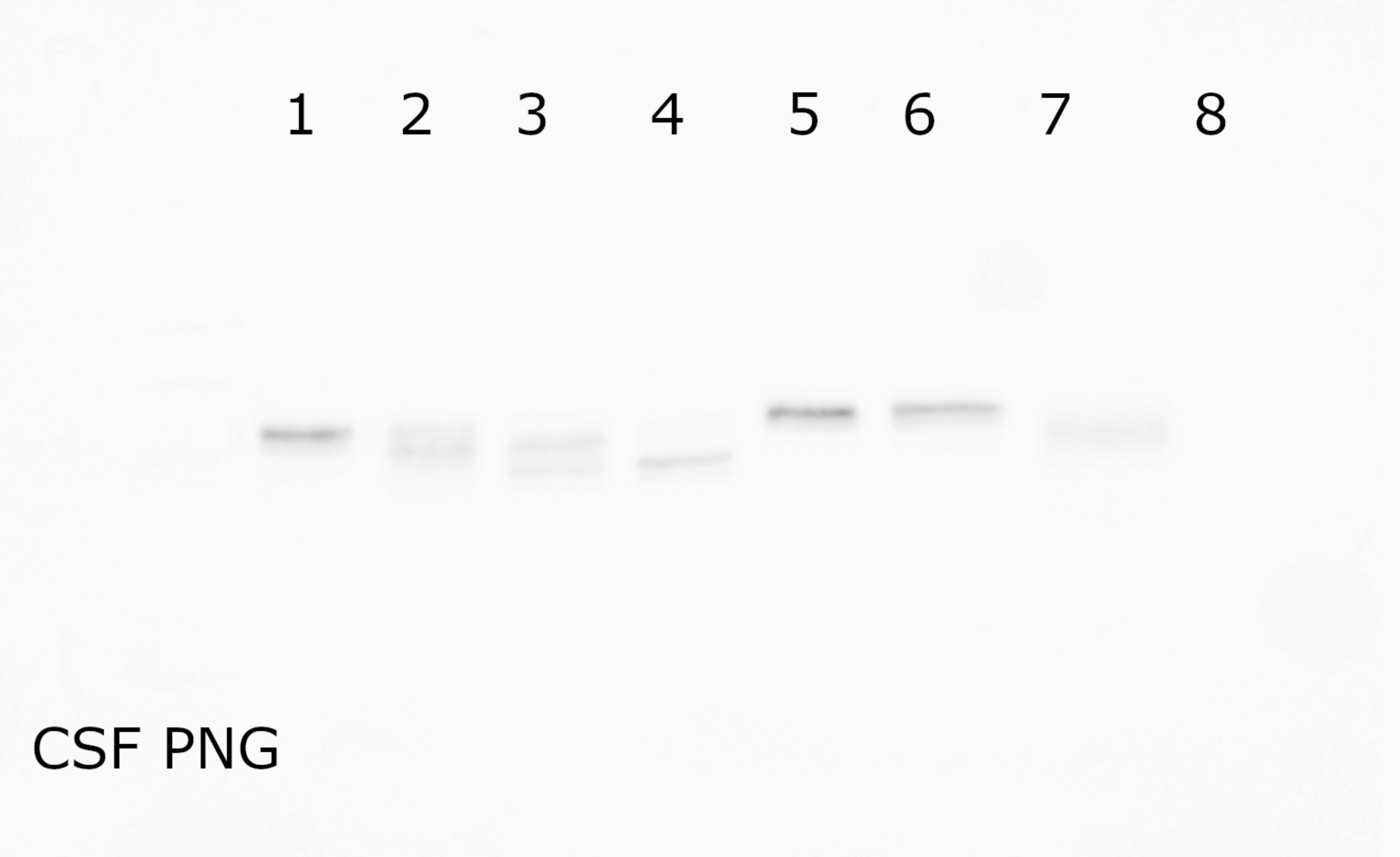

1	2	3	4	5	6	7	8	9	10	11	12	13	14	15	16	17
S31 P	M	S30	S30 P	S17	S17 P	S26	S26 P	S16	S16 P	S56	S56 P	S27	S27 P	S53	S53 P	S31

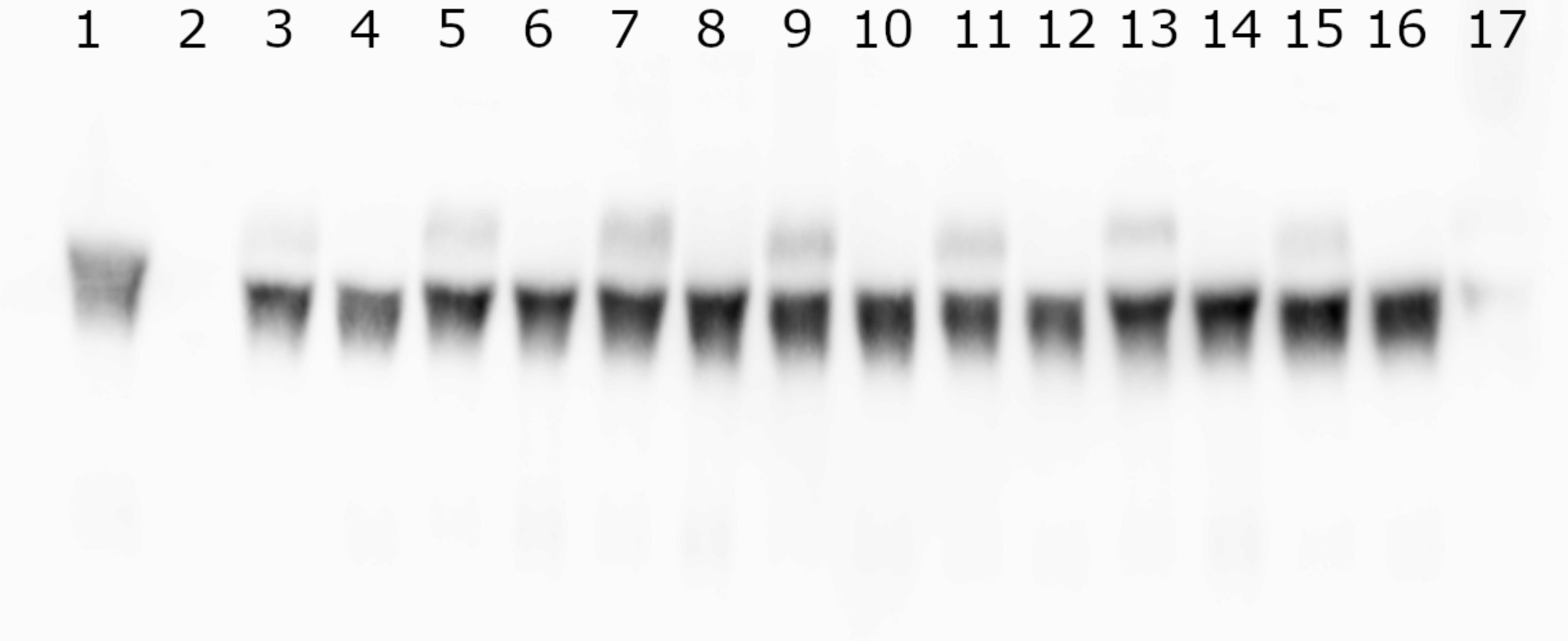

S1 phosph

1	2	3	4	5	6	7	8	9	10	11	12	13	14	15	16	17
M	S11	S11 P	S36	S36 P	S65	S65 P	S2	S2 P	CSF 11	CSF11 P	CSF36	CSF36 P	CSF65	CSF65 P	CSF2	CSF2 P

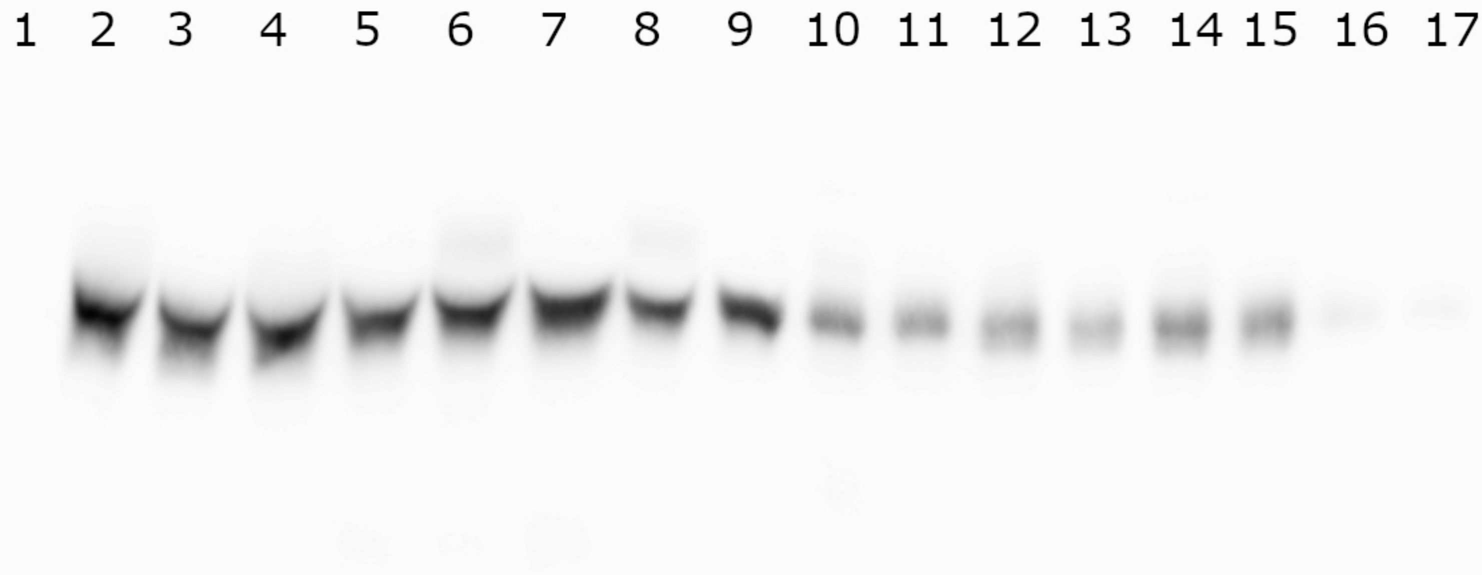

SCSF1 phosph

**1**	**2**	**3**	**4**	**5**	**6**	**7**	**8**	**9**	**10**	**11**	**12**	**13**	**14**	**15**	**16**	**17**
M	S11	S11 P	S36	S36 P	S65	S65 P	S2	S2 P	CSF 11	CSF11 P	CSF36	CSF36 P	CSF65	CSF65 P	CSF2	CSF2 P

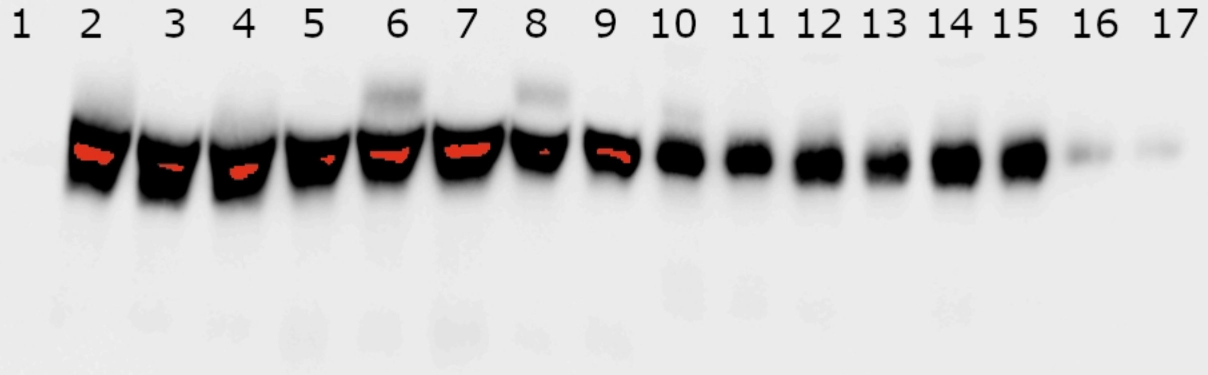

SCSF1 phosph - higher contrast

1	2	3	4	5	6	7	8	9	10	11	12	13	14	15	16	17
M	S4	S4 P	S60	S60 P	S37	S37 P	S42	S42 P	S10	S10 P	S6	S6 P	S51	S51 P	S70	S70 P

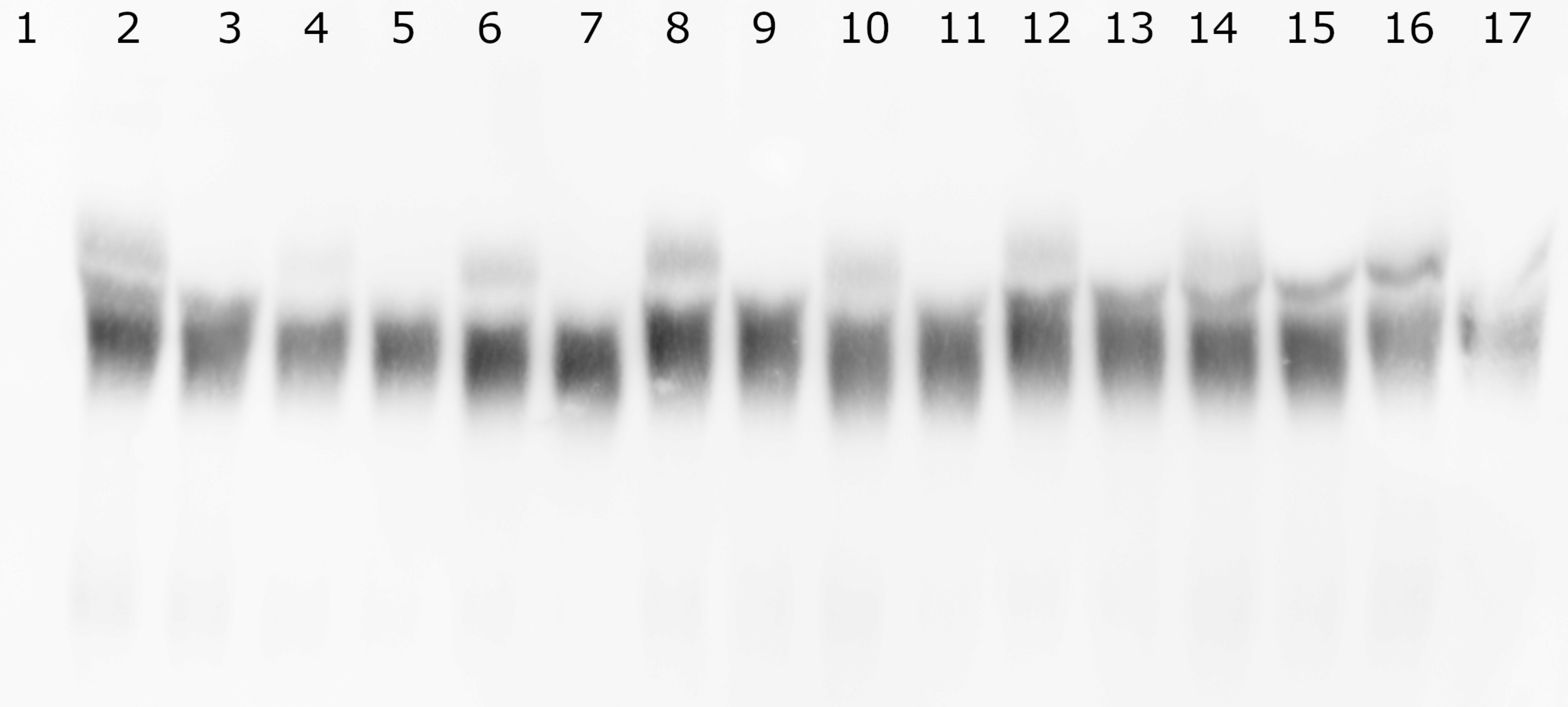

S2 phosph

1	2	3	4	5	6	7	8	9	10	11	12	13	14	15	16	17
M	S1	S1 P	S3	S3 P	S7	S7 P	S9	S9 P	S12	S12 P	S22	S22 P	S25	S25 P	S32	S32 P

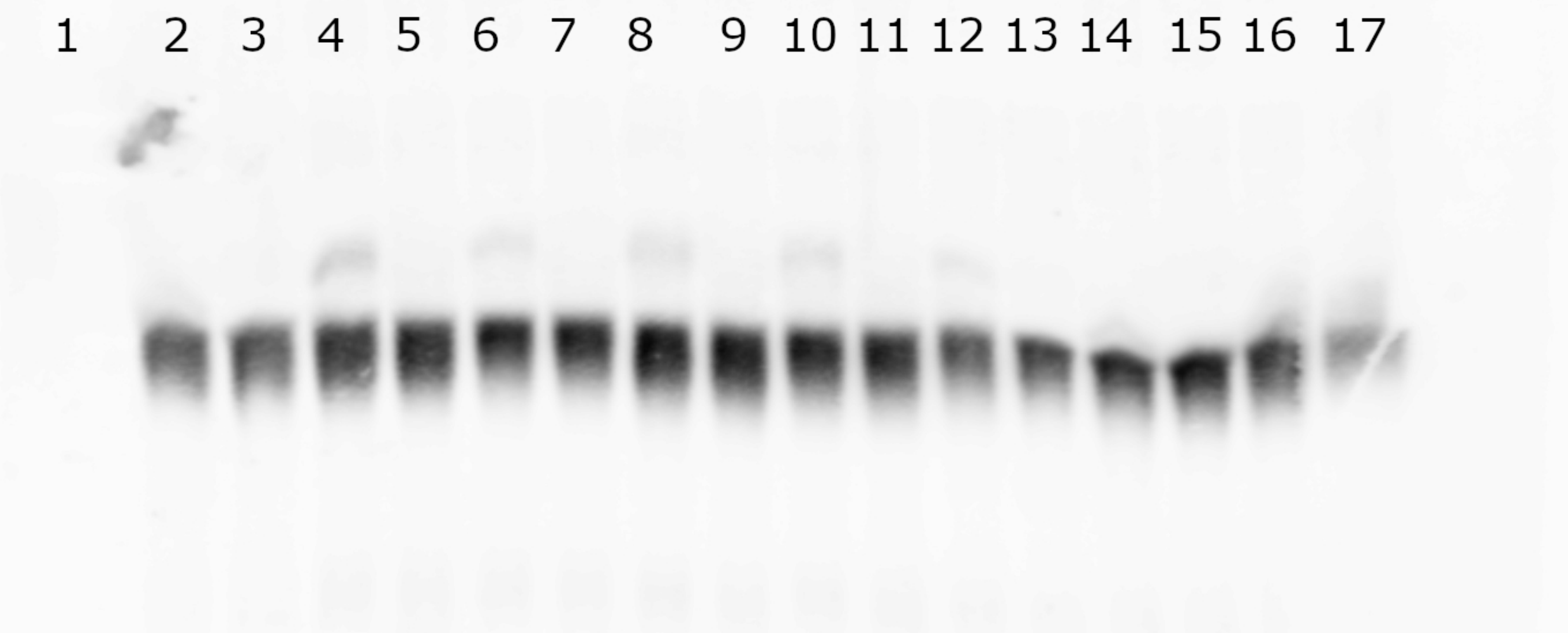

S3 phosph

1	2	3	4	5	6	7	8	9	10	11	12	13	14	15	16	17
M	S33	S33 P	S35	S35 P	S40	S40 P	S41	S41 P	S45	S45 P	S47	S47 P	S50	S50 P	S55	S55 P

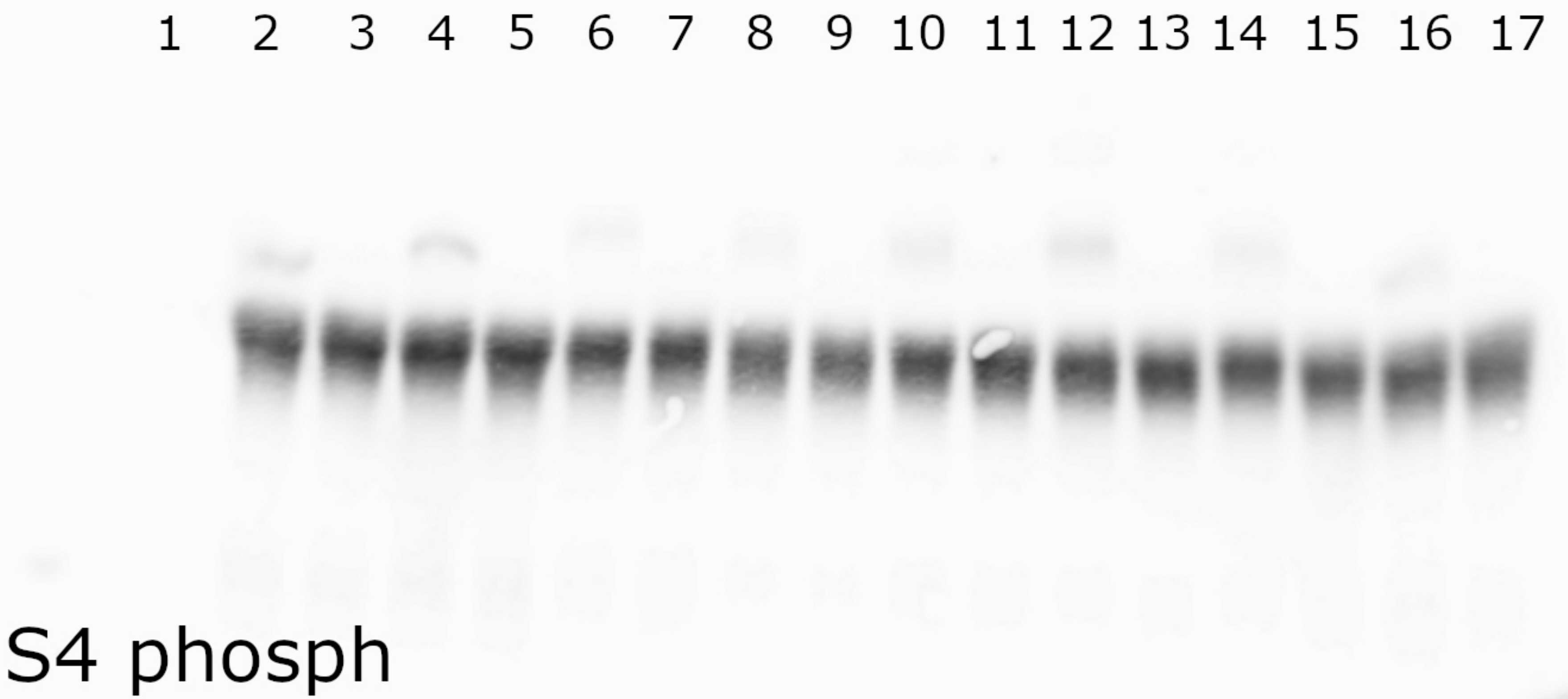

1	2	3	4	5	6	7	8	9	10	11	12	13	14	15	16	17
M	S33	S33 P	S35	S35 P	S40	S40 P	S41	S41 P	S45	S45 P	S47	S47 P	S50	S50 P	S55	S55 P

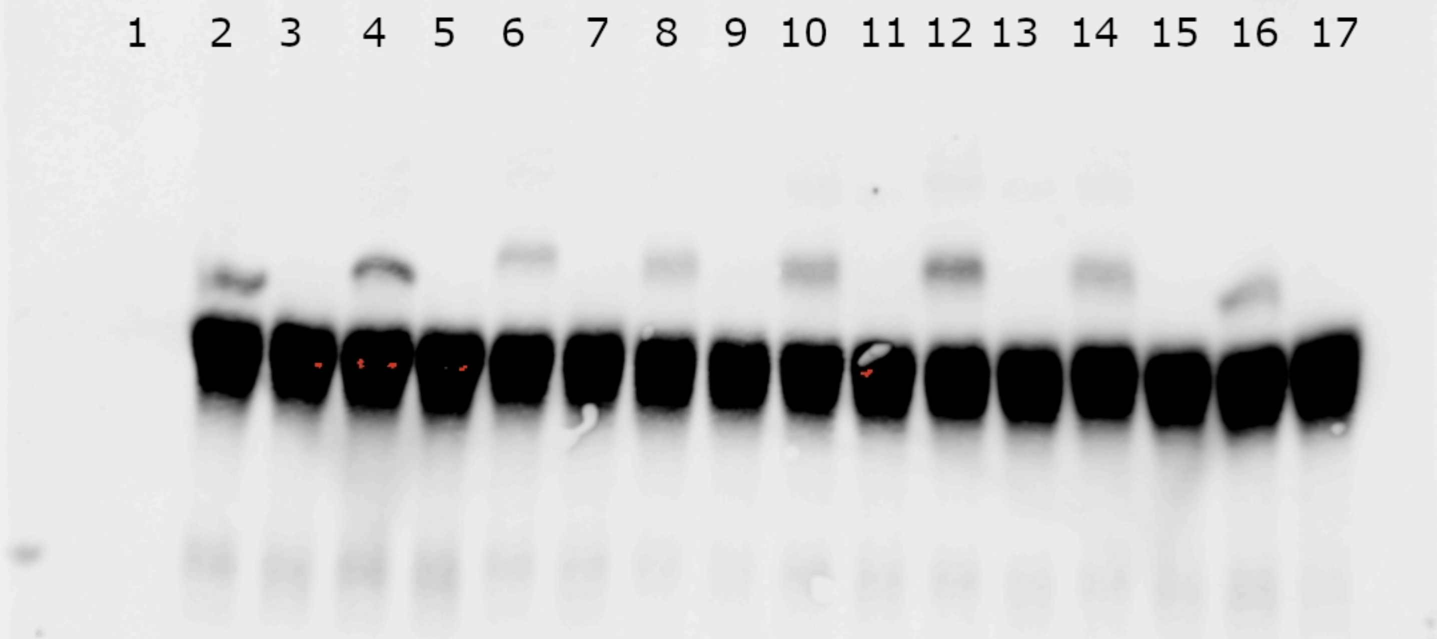

S4 phosph - higher contrast

1	2	3	4	5	6	7	8	9	10	11	12	13
M	S57	S57 P	S61	S61 P	S62	S62 P	S63	S63 P	S66	S66 P	S69	S69 P

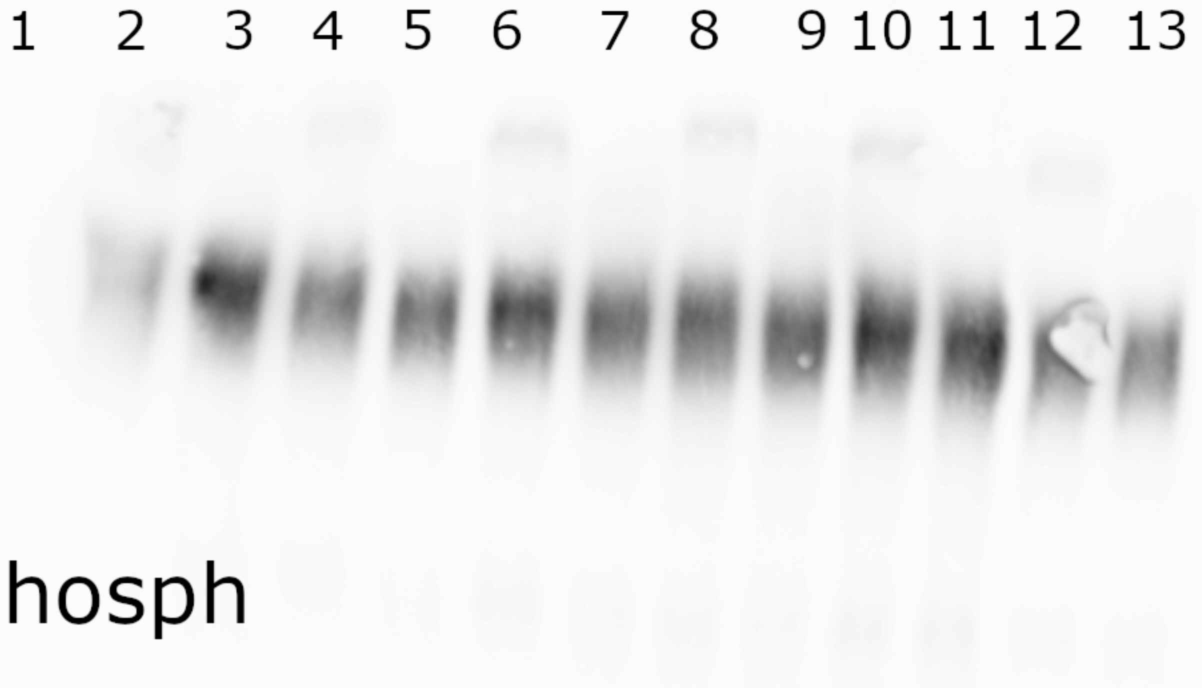

S5 phosph

1	2	3	4	5	6	7	8	9	10	11	12	13
M	S57	S57 P	S61	S61 P	S62	S62 P	S63	S63 P	S66	S66 P	S69	S69 P

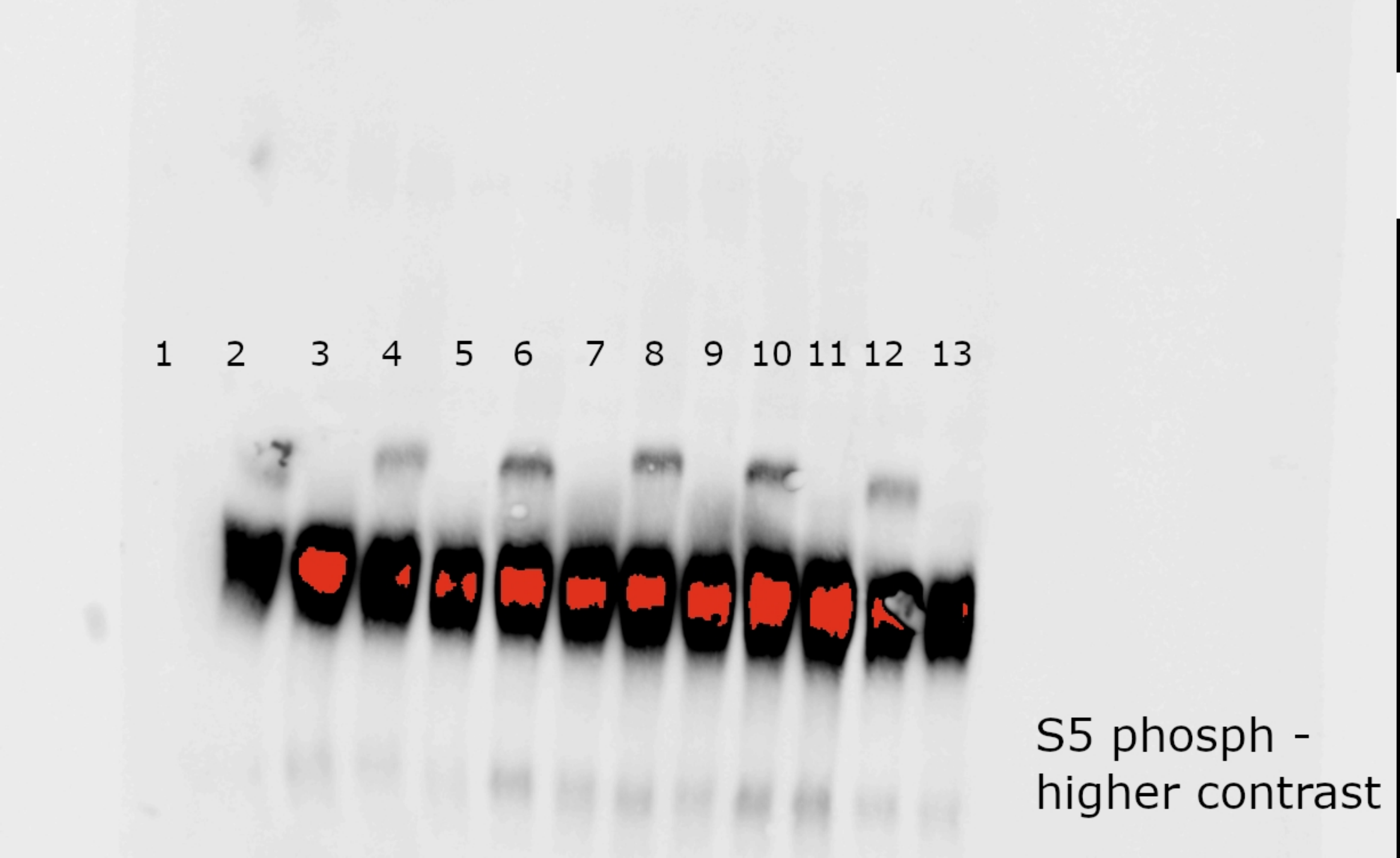

1	2	3	4	5	6	7	8	9	10	11	12	13	14	15	16	17
S62	S62 P	S55	S55 P	S1	S1 P	S33	S33 P	STA	S32	S32 P	S47	S47 P	S50	S50 P	S25	S25 P

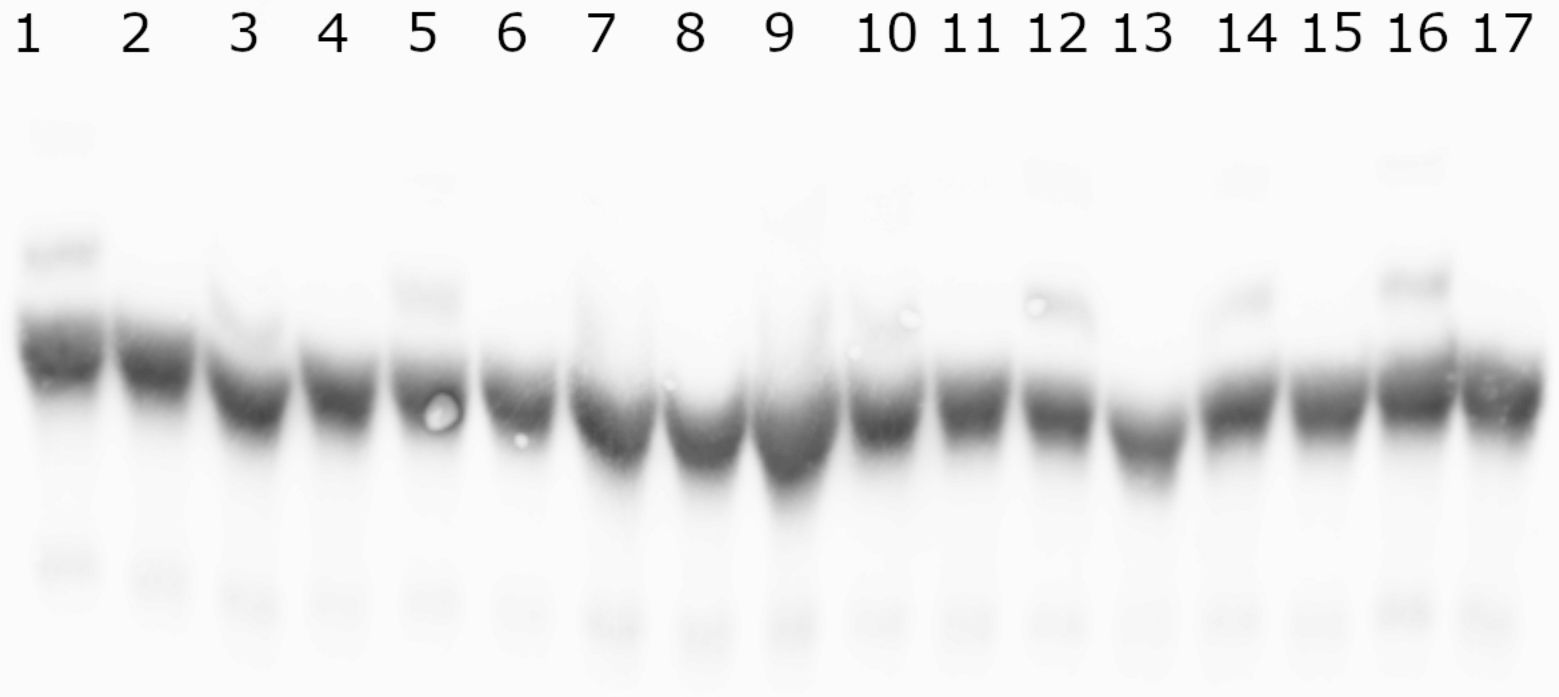

S6 phosph

1	2	3	4	5	6	7	8	9	10	11	12	13	14	15	16	17
S3	S3 P	S40	S40 P	S63	S63P	S57	S57 P	STA	S41	S41 P	S35	S35 P	S69	S69 P	S7	S7 P

1 2 3 4 5 6 7 8 9 10 11 12 13 14 15 16 17

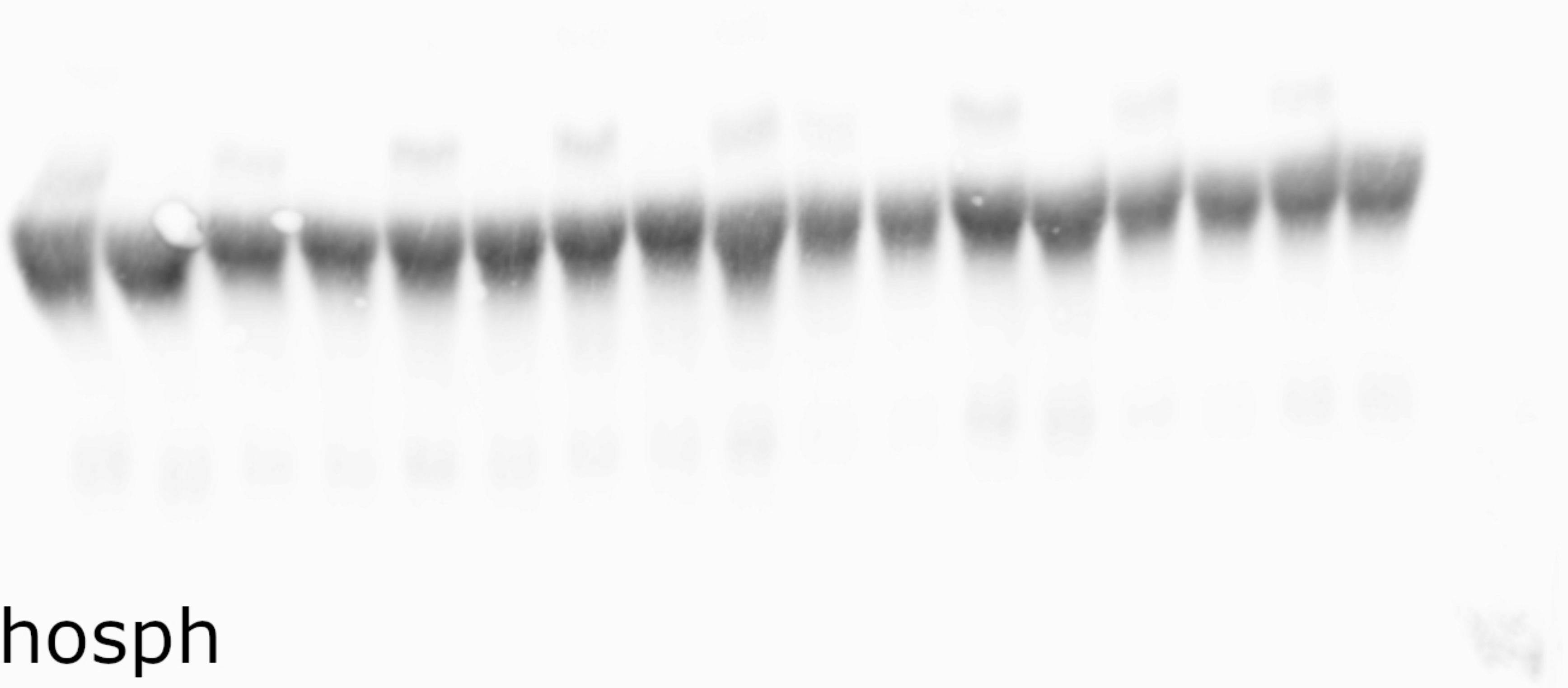

S7 phosph

1	2	3	4	5	6	7	8	9	10	11	12	13	14	15	16	17
S3	S3 P	S40	S40 P	S63	S63P	S57	S57 P	STA	S41	S41 P	S35	S35 P	S69	S69 P	S7	S7 P

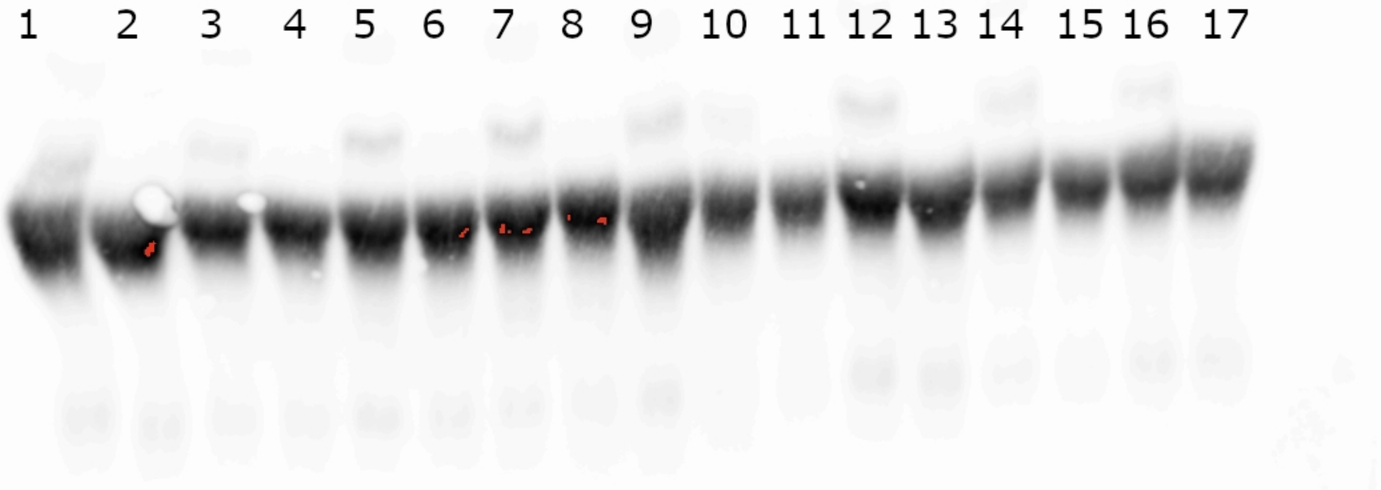

S7 phosph - higher contrast

1	2	3	4	5	6	7	8	9	10	11
S12	S12P	S66	S66 P	S22	S22 P	S45	S45 P	STA	S61	S61 P

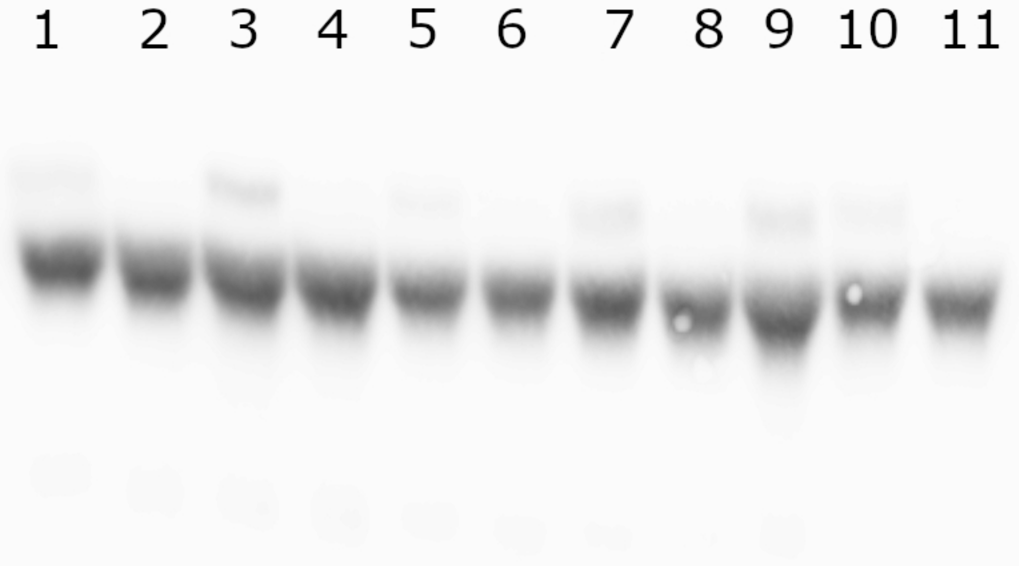

S8 phosph

1	2	3	4	5	6	7	8	9	10	11
S12	S12P	S66	S66 P	S22	S22 P	S45	S45 P	STA	S61	S61 P

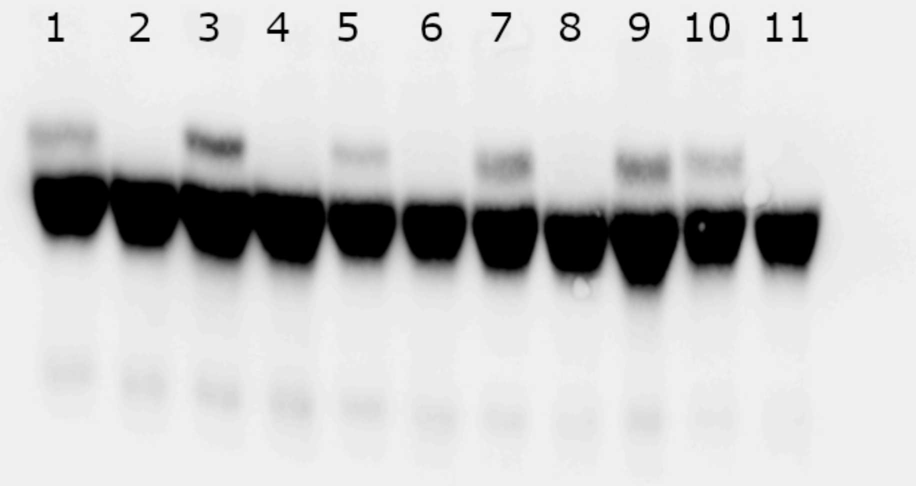

S8 phosph - higher contrast

1	2	3	4	5	6	7	8	9	10	11	12	13	14	15	16	17
M	CSF30	CSF30 P	CSF17	CSF17 P	CSF26	CSF26 P	CSF16	CSF16 P	CSF56	CSF56 P	CSF27	CSF27 P	CSF53	CSF53 P	CSF31	CSF31 P

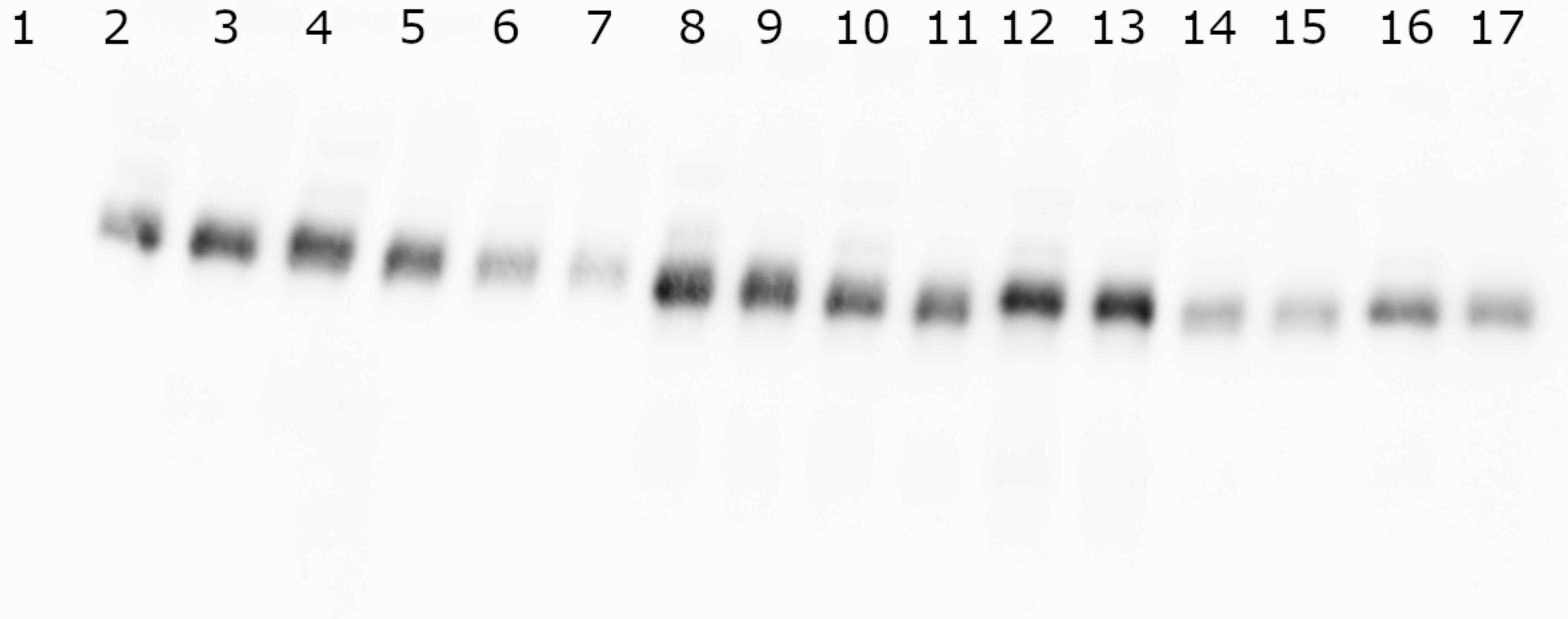

CSF1 phosph

1	2	3	4	5	6	7	8	9	10	11	12	13	14	15	16	17
M	CSF30	CSF30 P	CSF17	CSF17 P	CSF26	CSF26 P	CSF16	CSF16 P	CSF56	CSF56 P	CSF27	CSF27 P	CSF53	CSF53 P	CSF31	CSF31 P

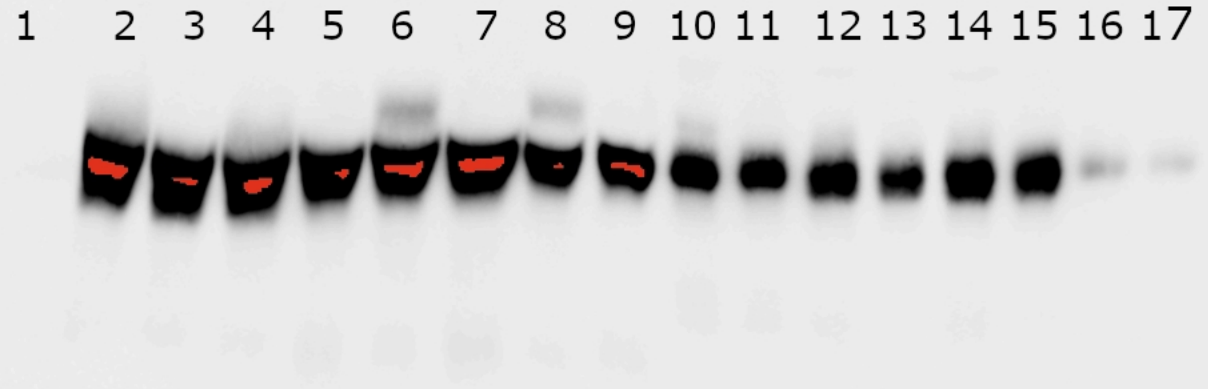

CSF1 phosph - higher contrast

1	2	3	4	5	6	7	8	9	10	11	12	13	14	15	16	17
M	CSF4	CSF4P	CSF60	CSF60 P	CSF37	CSF37 P	CSF42	CSF42 P	CSF10	CSF10 P	CSF6	CSF6 P	CSF51	CSF51 P	CSF70	CSF70 P

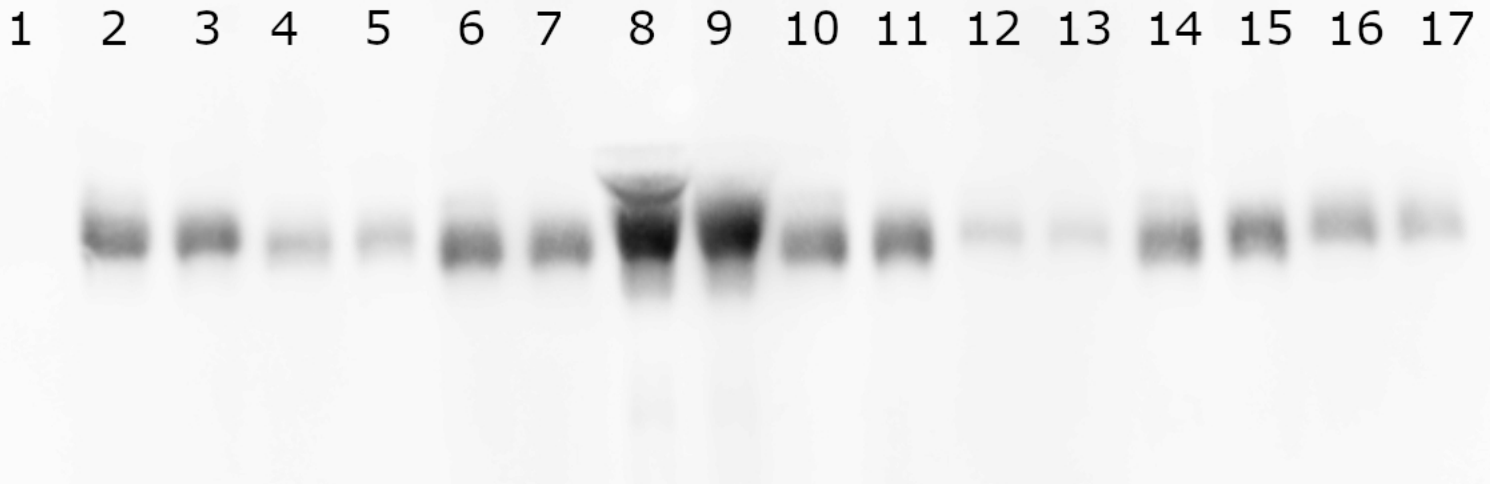

CSF2 phosph

1	2	3	4	5	6	7	8	9	10	11	12	13	14	15	16	17
M	CSF1	CSF1 P	CSF3	CSF3 P	CSF7	CSF7 P	CSF9*	CSF9 P*	CSF12	CSF12 P	CSF22	CSF22 P	CSF25	CSF25 P	CSF32	CSF32 P

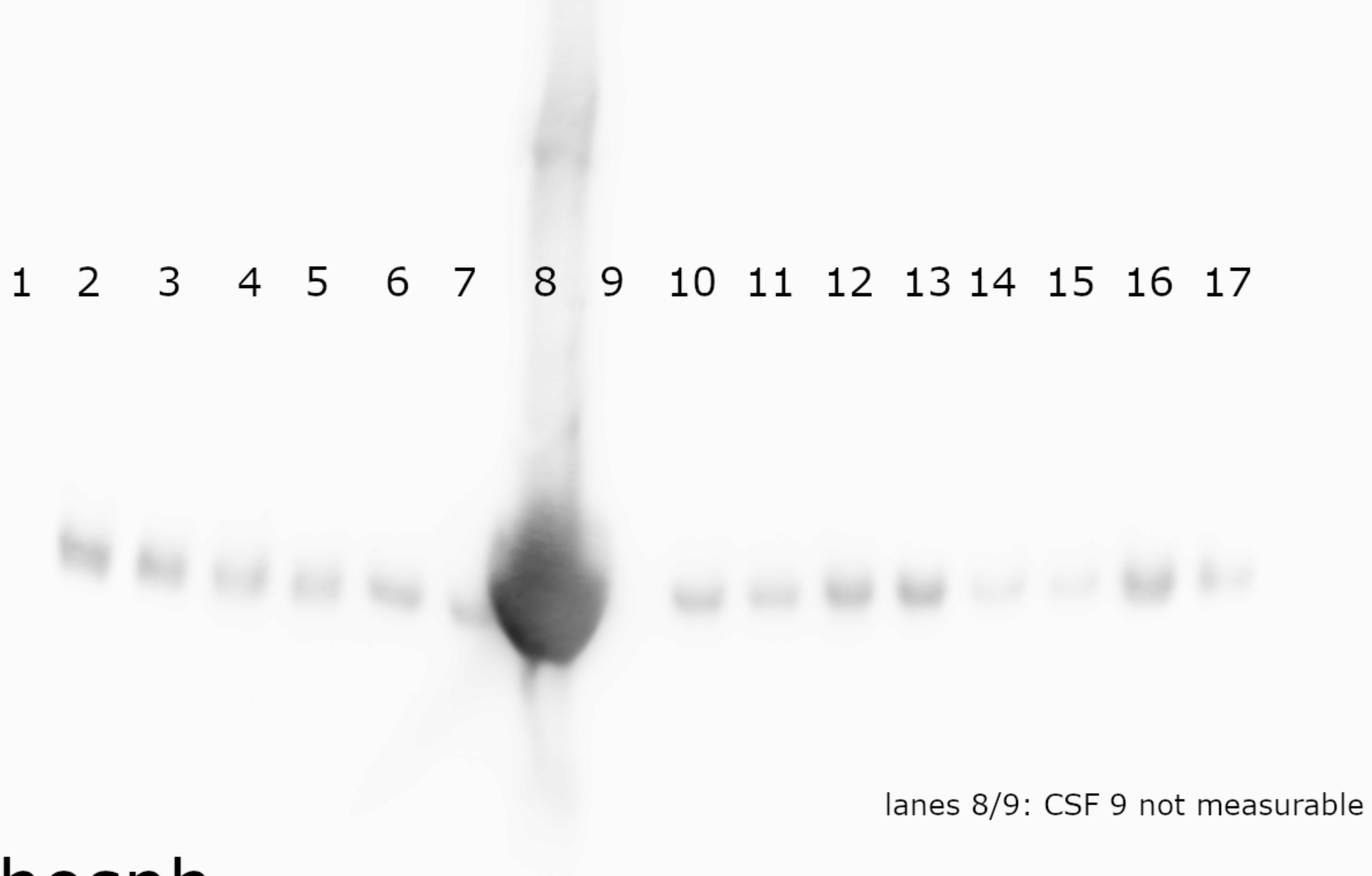

lanes 8/9: CSF 9 not measurable

CSF3 phosph

1	2	3	4	5	6	7	8	9	10	11	12	13	14	15	16	17
M	CSF33	CSF33 P	CSF35	CSF35 P	CSF40	CSF40 P	CSF41	CSF41 P	CSF45	CSF45 P	CSF47	CSF47 P	CSF50	CSF50 P	CSF55	CSF55 P

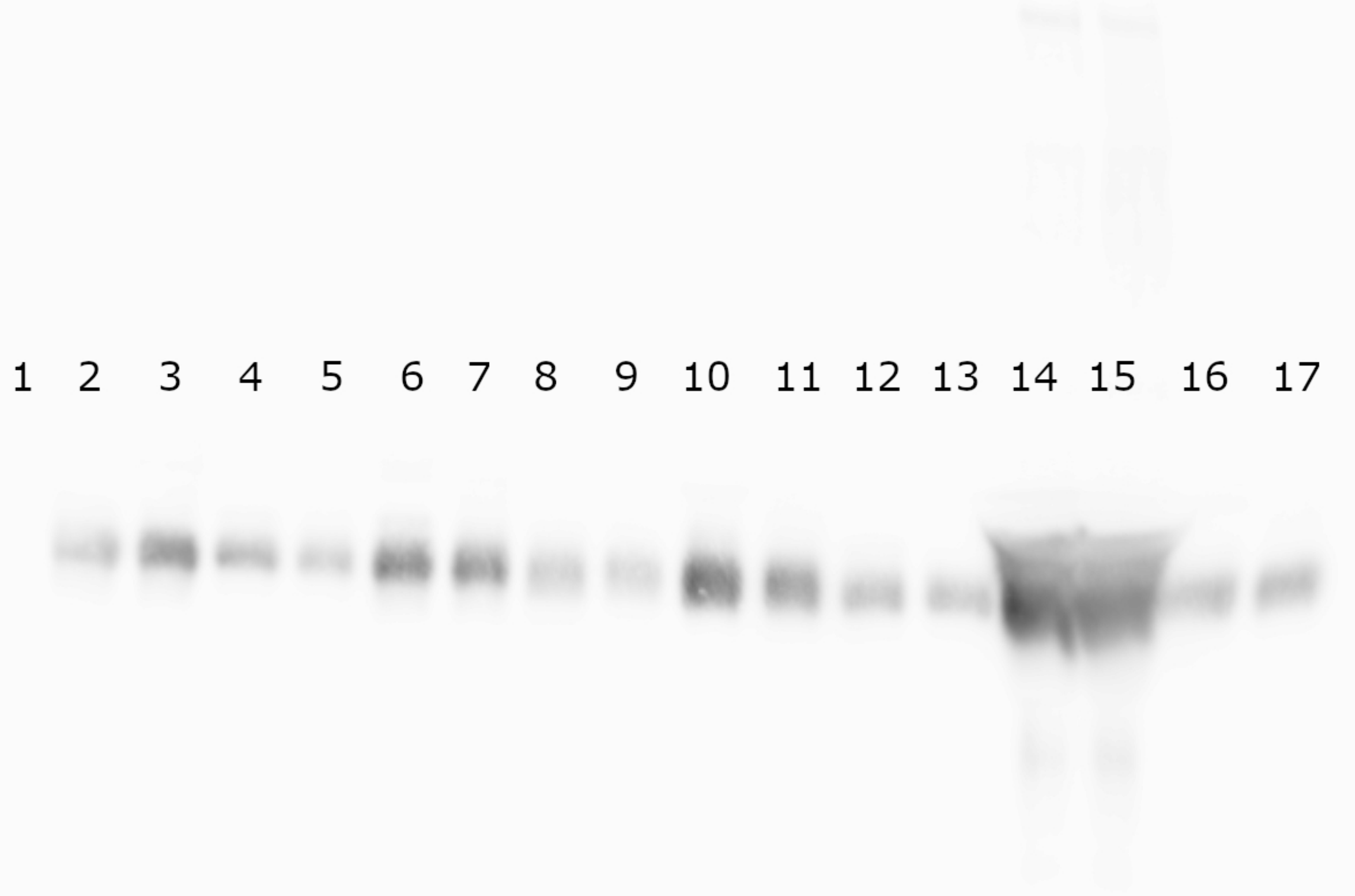

CSF4 phosph

1	2	3	4	5	6	7	8	9	10	11	12	13	14	15	16	17
M	CSF33	CSF33 P	CSF35	CSF35 P	CSF40	CSF40 P	CSF41	CSF41 P	CSF45	CSF45 P	CSF47	CSF47 P	CSF50	CSF50 P	CSF55	CSF55 P

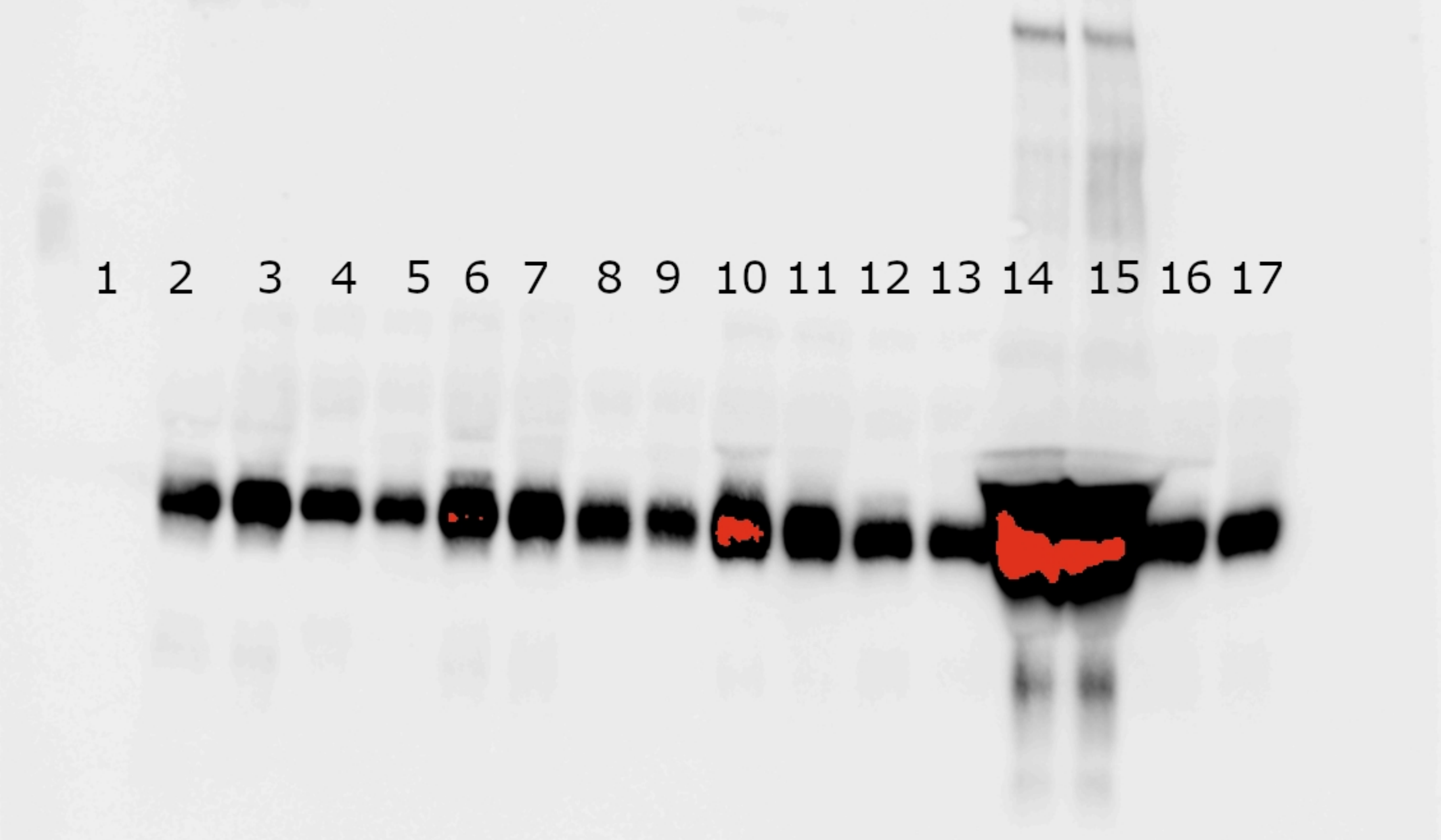

CSF4 phosph - higher contrast

1	2	3	4	5	6	7	8	9	10	11	12	13	14	15	16	17
CSF62	CSF62 P	CSF55	CSF55 P	CSF1	CSF1 P	CSF33	CSF33 P	STA	CSF32	CSF32 P	CSF47	CSF47 P	CSF50	CSF50 P	CSF25	CSF25 P

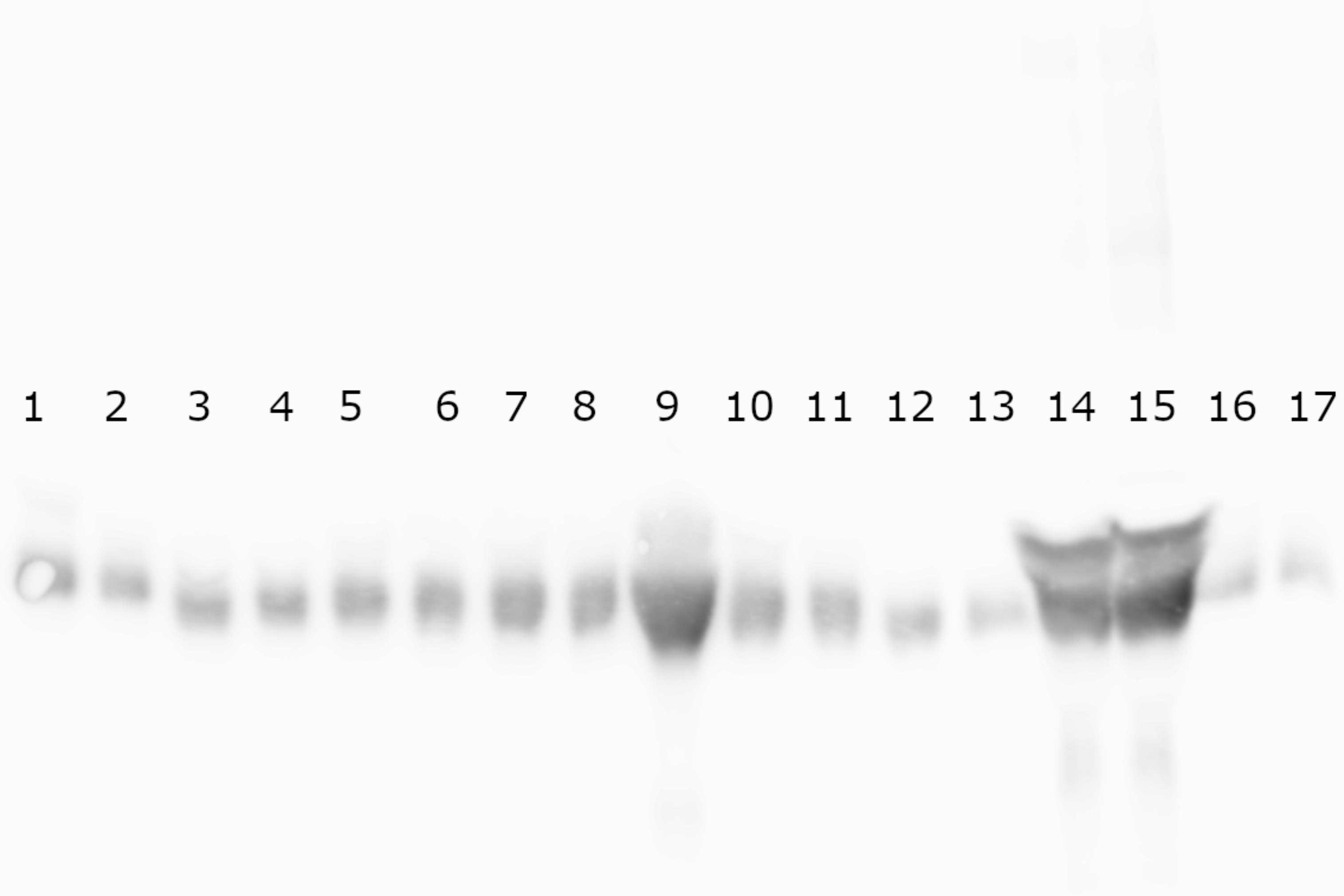

CSF5 phosph

1	2	3	4	5	6	7	8	9	10	11	12	13	14	15	16	17
CSF62	CSF62 P	CSF55	CSF55 P	CSF1	CSF1 P	CSF33	CSF33 P	STA	CSF32	CSF32 P	CSF47	CSF47 P	CSF50	CSF50 P	CSF25	CSF25 P

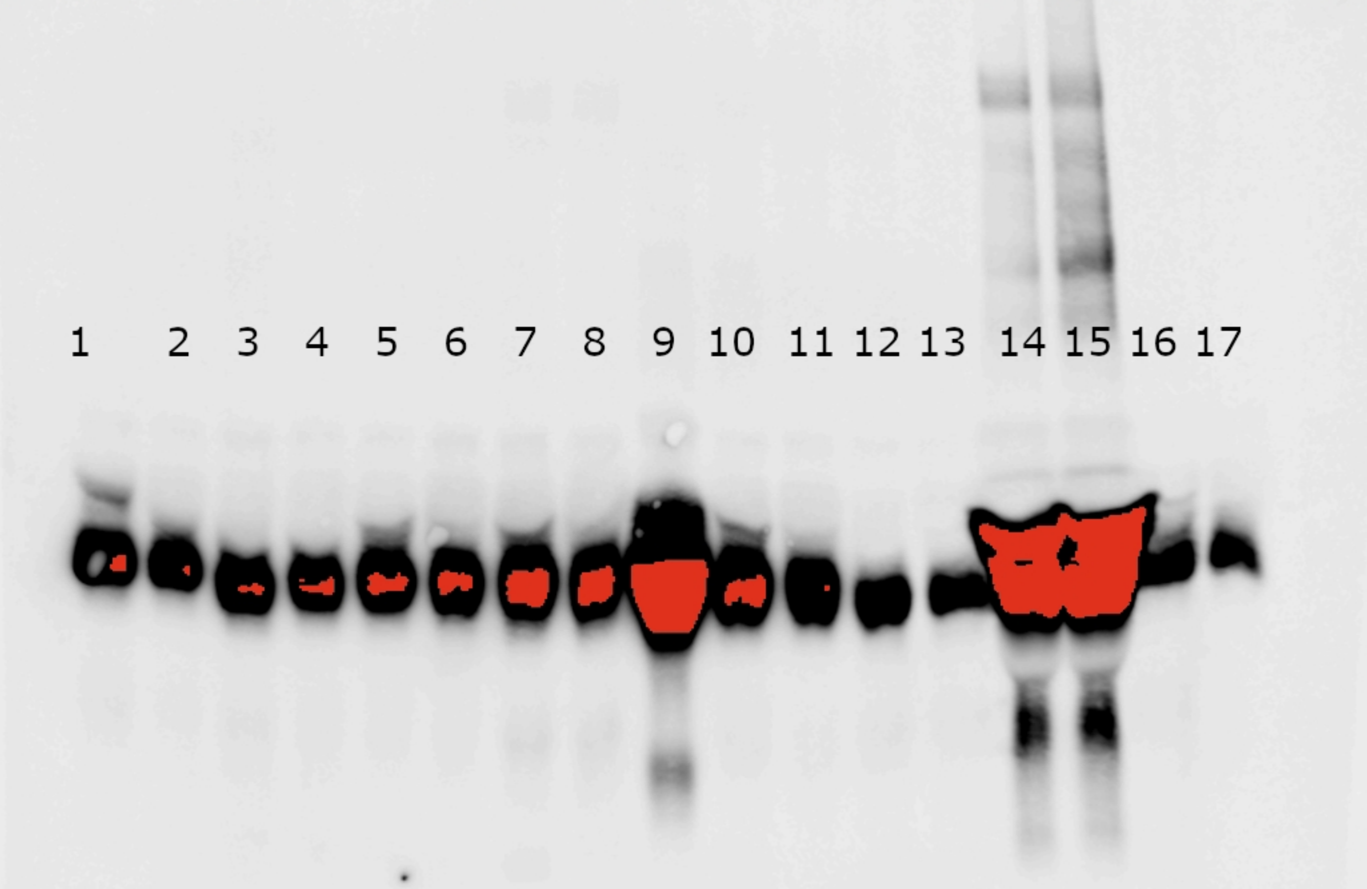

CSF5 phosph - higher contrast

1	2	3	4	5	6	7	8	9	10	11	12	13	14	15	16	17
CSF3	CSF3 P	CSF40	CSF40 P	CSF63	CSF63 F	CSF57	CSF57 P	STA	CSF41	CSF41 P	CSF35	CSF35 P	CSF69	CSF69 P	CSF7	CSF7 P

1 2 3 4 5 6 7 8 9 10 11 12 13 14 15 16 17

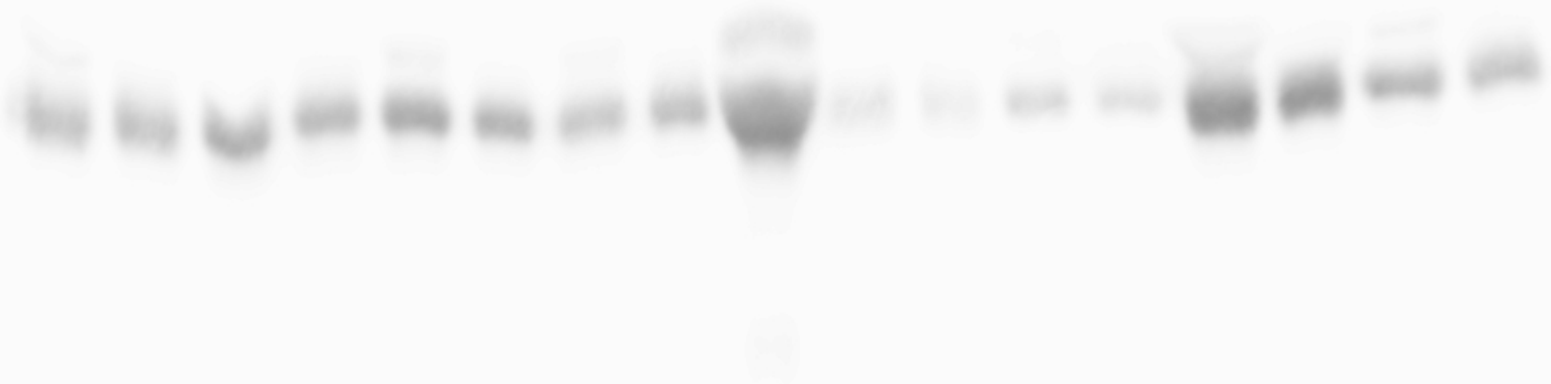

CSF6 phosph

1	2	3	4	5	6	7	8	9	10	11	12	13	14	15	16	17
CSF3	CSF3 P	CSF40	CSF40 P	CSF63	CSF63 [illegible]	CSF57	CSF57 P	STA	CSF41	CSF41 P	CSF35	CSF35 P	CSF69	CSF69 P	CSF7	CSF7 P

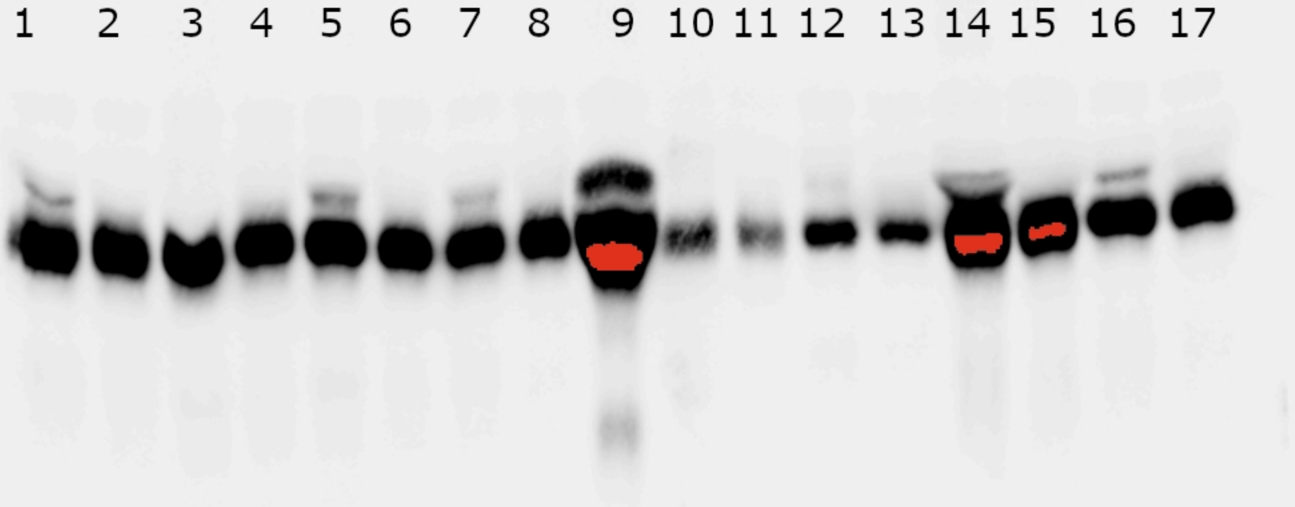

CSF6 phosph - higher contrast

1	2	3	4	5	6	7	8	9	10	11
CSF12	CSF12 P	CSF66	CSF66 P	CSF22	CSF22 [illegible]	CSF45	CSF45 P	STA	CSF61	CSF61 P

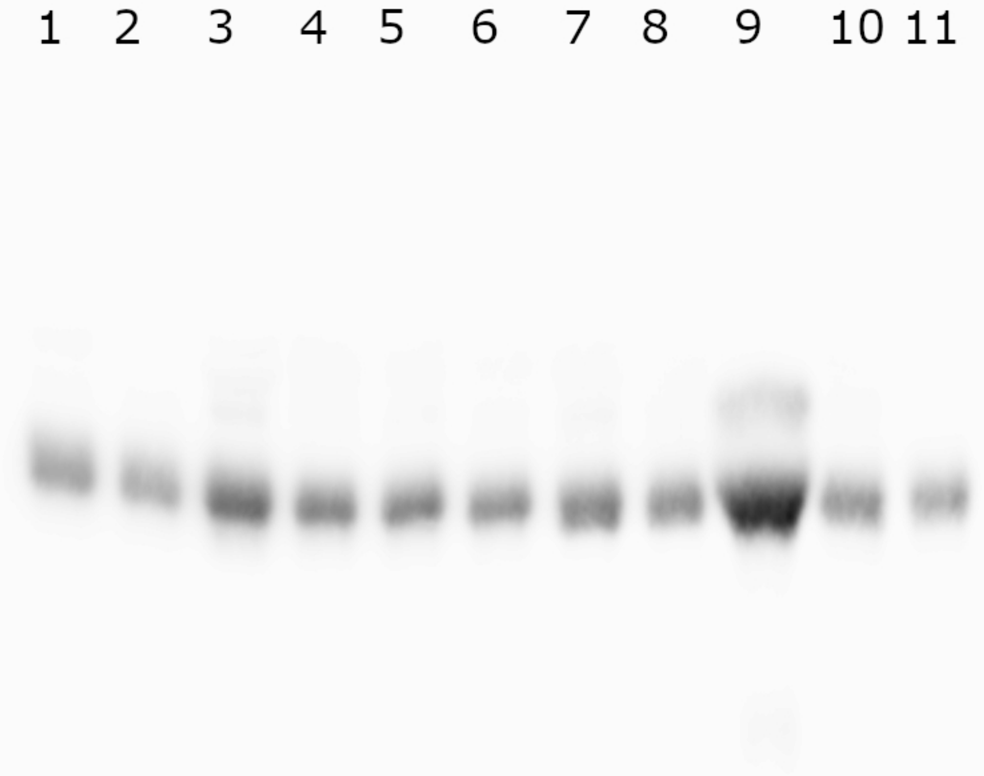

CSF7 phosph

1	2	3	4	5	6	7	8	9	10	11
CSF12	CSF12 P	CSF66	CSF66 P	CSF22	CSF22 P	CSF45	CSF45 P	STA	CSF61	CSF61 P

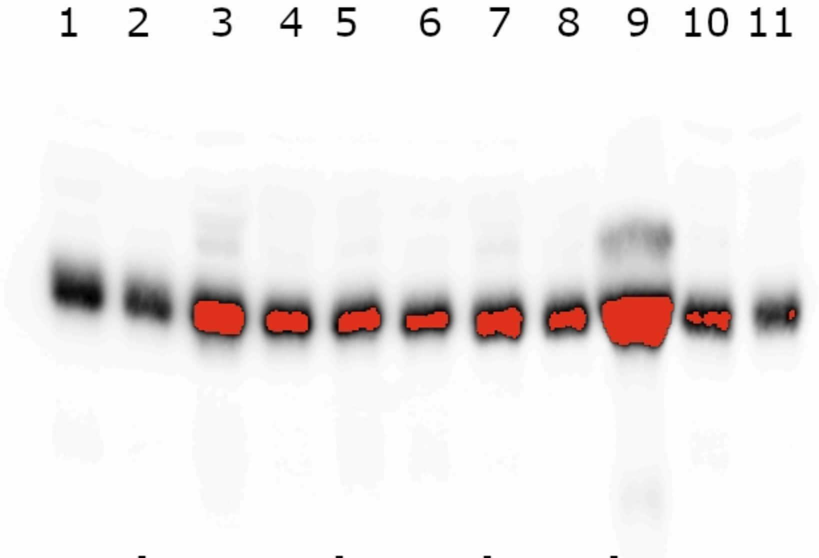

CSF7 phosph - higher contrast

1	2	3	4	5	6	7	8	9	10	11	12	13
M	CSF57	CSF57 P	CSF61	CSF61 P	CSF62	CSF62 P	CSF63	CSF63 P	CSF66	CSF66 P	CSF69	CSF69 P

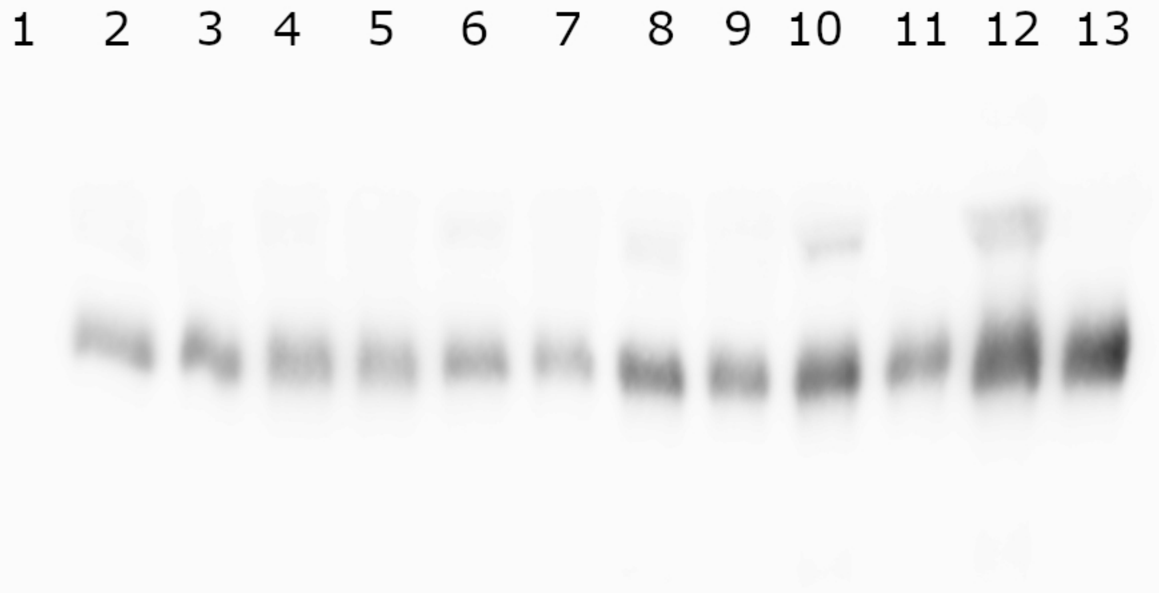

CSF8 phosph

1	2	3	4	5	6	7	8	9	10	11	12	13
M	CSF57	CSF57 P	CSF61	CSF61 P	CSF62	CSF62 P	CSF63	CSF63 P	CSF66	CSF66 P	CSF69	CSF69 P

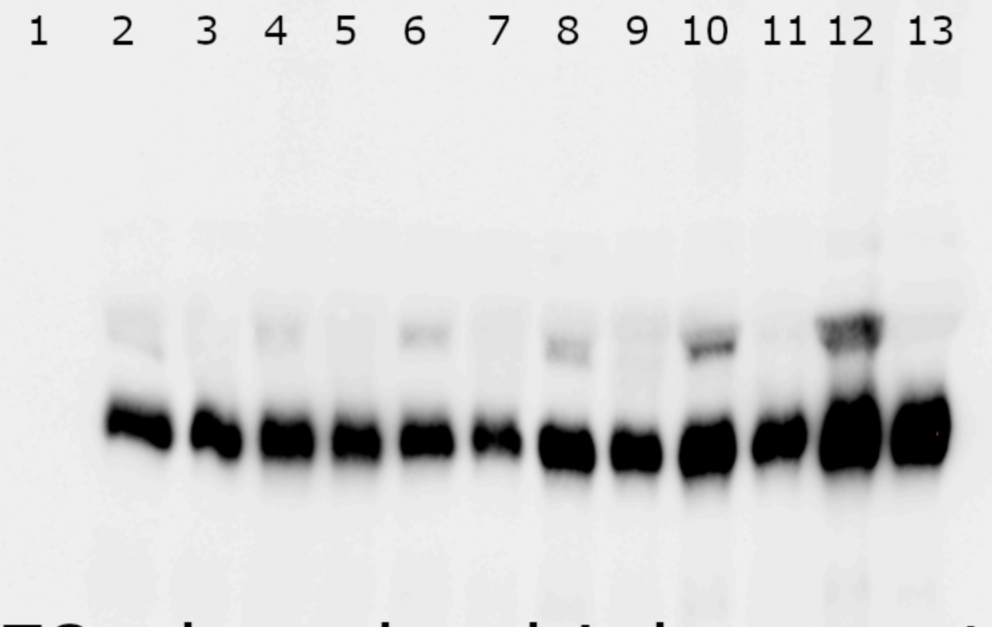

CSF8 phosph - higher contrast
